# USF Binding Sequences from the HS4 Insulator Element Impose Early Replication Timing on a Vertebrate Replicator

**DOI:** 10.1371/journal.pbio.1001277

**Published:** 2012-03-06

**Authors:** Vahideh Hassan-Zadeh, Sabarinadh Chilaka, Jean-Charles Cadoret, Meiji Kit-Wan Ma, Nicole Boggetto, Adam G. West, Marie-Noëlle Prioleau

**Affiliations:** 1 Institut Jacques Monod, Centre National de la Recherche Scientifique, Université Paris Diderot, Paris, France; 2 Institute of Cancer Sciences, College of Medical, Veterinary and Life Sciences, University of Glasgow, Glasgow, United Kingdom; National Cancer Institute, United States of America

## Abstract

The nuclear genomes of vertebrates show a highly organized program of DNA replication where GC-rich isochores are replicated early in S-phase, while AT-rich isochores are late replicating. GC-rich regions are gene dense and are enriched for active transcription, suggesting a connection between gene regulation and replication timing. Insulator elements can organize independent domains of gene transcription and are suitable candidates for being key regulators of replication timing. We have tested the impact of inserting a strong replication origin flanked by the β-globin HS4 insulator on the replication timing of naturally late replicating regions in two different avian cell types, DT40 (lymphoid) and 6C2 (erythroid). We find that the HS4 insulator has the capacity to impose a shift to earlier replication. This shift requires the presence of HS4 on both sides of the replication origin and results in an advance of replication timing of the target locus from the second half of S-phase to the first half when a transcribed gene is positioned nearby. Moreover, we find that the USF transcription factor binding site is the key *cis*-element inside the HS4 insulator that controls replication timing. Taken together, our data identify a combination of *cis*-elements that might constitute the basic unit of multi-replicon megabase-sized early domains of DNA replication.

## Abstract 2

A combination of *cis*-regulatory elements can impose the formation of an early replicating domain in a naturally late replicating region and might constitute the basic unit of early replicating domains.

## Author Summary

All eukaryotic organisms must duplicate their genome precisely once before cell division. This occurs according to an established temporal program during S-phase (when DNA synthesis takes place) of the cell cycle. In vertebrates, this program is regulated at the level of large chromosomal domains ranging from 200 kb to 2 Mb, but the molecular mechanisms that control the temporal firing order of animal replication origins are not clearly understood. Using the genetically tractable chicken DT40 cell system, we identified a minimal combination of *cis*-regulatory DNA elements that is able to shift the timing of a naturally “mid-late” replicated region to “mid-early.” This critical group of elements is composed of one strong replication origin flanked by binding sequences for the upstream stimulatory factor (USF) protein. The additional presence of a strongly transcribed gene shifted the region towards an even earlier replication time, suggesting cooperation between *cis*-elements when establishing temporal programs of replication. We speculate that USF binding sequences cooperate with sites of replication initiation and transcribed genes to promote the establishment of early replicating domains along vertebrate genomes.

## Introduction

The nuclear genome of higher eukaryotes is replicated according to an established temporal program. Although replication timing has been precisely analyzed in many cell types from several organisms, the molecular mechanisms involved in its regulation are still poorly characterized [1]. It is now well established that there is an important correlation between replication timing and gene regulation with GC-rich and gene-rich regions replicating early and AT-rich and gene-poor regions replicating late [2]–[4]. However, two recent genome-wide studies in mouse and *Drosophila* show that this relationship is indirect, since a large fraction of late-replicating genes (about 20%) are expressed and some genes change transcription without change in replication timing and vice versa [5],[6]. The simplest explanation is that replication timing is related to chromosomal organization rather than transcription. Several studies suggest that post-translational histone modifications directly regulate replication timing, but the effects of altering particular modifications are relatively minor in vertebrates [7],[8]. The *cis* tethering of histone acetyltransferase activity adjacent to the human *β-globin* origin of replication induced only a weak shift in replication timing from late to mid-late S-phase in lymphocytes, suggesting that this signal is insufficient to organize the early domain of replication that exists in erythroid cells [7]. The disruption of histone modifications *in trans* following the genetic knockout of histone modifying enzymes also results in minor changes in replication timing. Only a minority (5/23) of 23 single copy loci displayed a weak shift of replication timing in a panel of mutant mouse ES cell lines that were disrupted for histone deacetylation; H3K4, H3K9, and H3K27 methylation; or DNA methylation activities [9]. Depletion of H3K9me2 has no effect on replication timing genome-wide [10]. A direct role for histone modifications in the regulation of early replication timing remains to be demonstrated in vertebrates. One hypothesis is that they may represent a secondary, fine-tuning role [11]. In contrast, origin efficiency is not dependent on canonical histone marks such as H3 or H4 acetylation or di- or trimethylation of H3K4 in vertebrates [12]–[17]. However, it was shown that histone acetylation is critical for replication origin activation at the amplified *Drosophila chorion* locus and that insulator elements protect amplification of this origin from position effect, suggesting that some specific origins might be controlled by histone tails modifications [18],[19].

In general, euchromatic domains reside in the interior of the nucleus and replicate in early S-phase, whereas heterochromatic domains localize to the nuclear periphery or near nucleoli and replicate late [20]. In every case examined, the dynamic changes in replication timing observed during development are accompanied by sub-nuclear repositioning [5],[21]. Moreover, replication timing is re-established during early G1-phase at the timing decision point (TDP), coincident with the repositioning of chromosomal domains in the nucleus after mitosis [22]–[24]. Therefore, the molecular events that direct the organization of discrete chromosomal domains are also predicted to play an important role in regulating replication timing. The *cis*-acting DNA elements that organize such genomic domains, particularly those that are replicated in the first half of S-phase, remain to be determined.

Insulator elements have been found to set the boundaries of transcriptionally active and repressive chromatin domains and represent candidate regulators of early DNA replication domain organization [25]–[27]. Insulators can be used in a transgenic context to shield transgenes from chromosomal position effect, where the chromatin environment around an integration site can dominantly silence or enhance transgene expression. The majority of random integration sites in vertebrates result in pervasive chromosomal silencing, but a minor fraction of loci are permissive to transgene expression, with a slight portion under the control of an endogenous enhancer. The biochemical activities that underlie the protection from these two types of regulatory interference are different, and the elements that are involved have distinct names: those that can block activation by enhancers are known as enhancer-blocking insulators, whereas those that protect against chromosomal silencing are known as barrier insulators.

The first insulator element to be identified in vertebrates is HS4, which lies at the 5′ boundary of the chicken *β-globin* domain [28]. A 275 bp compound HS4 element harbors separable enhancer-blocking and barrier activities [29]–[31]. HS4's enhancer-blocking activity is mediated by a single binding site (FII) for the ubiquitously expressed zinc finger protein CTCF [29]. HS4's barrier function depends on four transcription factor binding sites (FI, FIII, FIV, and FV) [31]. The FIV site is bound by the ubiquitous transcription factors USF1 and USF2, which recruit a panel of active histone modifications that interrupt the spread of repressive histone modifications [32]–[34]. The remaining barrier sites FI, FIII, and FV, each bound by the broadly expressed zinc finger protein VEZF1, mediate protection from DNA methylation [35].

Replication origins have been precisely mapped along the chicken *β-globin* locus in the early erythroid cell line 6C2 [17]. This region is replicated by a group of four origins located at the 5′ HS4 insulator, 5′ and 3′ of the *ρ-globin* gene, and inside the promoter of the *β^A^-globin* gene. The globin genes are not expressed in 6C2 cells, and only the replication origin overlapping the HS4 insulator is marked by active histone modifications [36]. Moreover, the chicken *β-globin* locus is replicated early in S-phase independently of globin gene expression in contrast to the paralogue human *β-globin* locus [17],[37]. In this study, we explored the possibility that the HS4 insulator may establish early DNA replication timing when inserted into a locus that is normally replicated in late or mid-late S-phase. To do so, we studied the effect of targeting insulator constructs on the timing of a mid-late replicating locus in DT40 lymphoid cells. We also studied the replication timing of insulator containing transgenes that are randomly integrated into erythroid cells.

Our data show that the introduction of the strong *β^A^-globin* replication origin flanked by HS4 insulator elements can significantly advance the replication timing of a mid-late replicating region. Surprisingly, the insulator activities of the HS4 element are not required for the imposition of earlier replication. We find that the USF protein binding site from the HS4 insulator is sufficient to control replication timing. Moreover, we demonstrate that USF binding sites need to flank the origin in order to drive a replication timing shift. The shift to earlier replication becomes dramatic when a transcriptionally active promoter is combined with HS4 insulators.

## Results

### Selection of a 100 kb Mid-Late Replicating Region That Is Devoid of Origins

**Figure 1:**
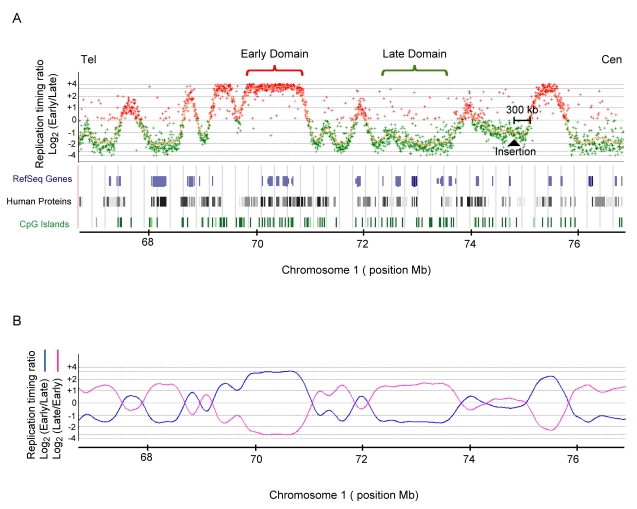
Genome-wide analysis of replication timing in DT40 cells. (A) Replication timing profile along a portion of Chromosome 1 is shown. This profile was obtained with cells sorted into three fractions (early, mid-, and late S-phase). After immunoprecipitation, BrdU pulse-labeled nascent DNA from early and late fractions was differentially labeled and cohybridized to a chicken whole-genome oligonucleotide microarray at a density of one probe every 5.6 kb. The log_2_-ratio (early/late) of the abundance of each probe in the early and late S-phase is shown. Early and late domains are in red and green, respectively. The chosen site of insertion is indicated. Below annotated genes, human proteins and CpG islands are shown. (B) Replicate experiments with nascent strands extracted from cells sorted into two fractions, early and late-replicating DNA, were reciprocally labeled (“dye-switch”) and hybridized. The log_2_-ratio timing profiles were smoothed using the Moving Average option of the Agilent Genomic Workbench 5.0 software with the linear algorithm and 200 kb windows (log_2_(Early/Late) blue and log_2_(Late/Early) pink). The mirror image shows the high degree of correlation between replicates.

**Figure 2:**
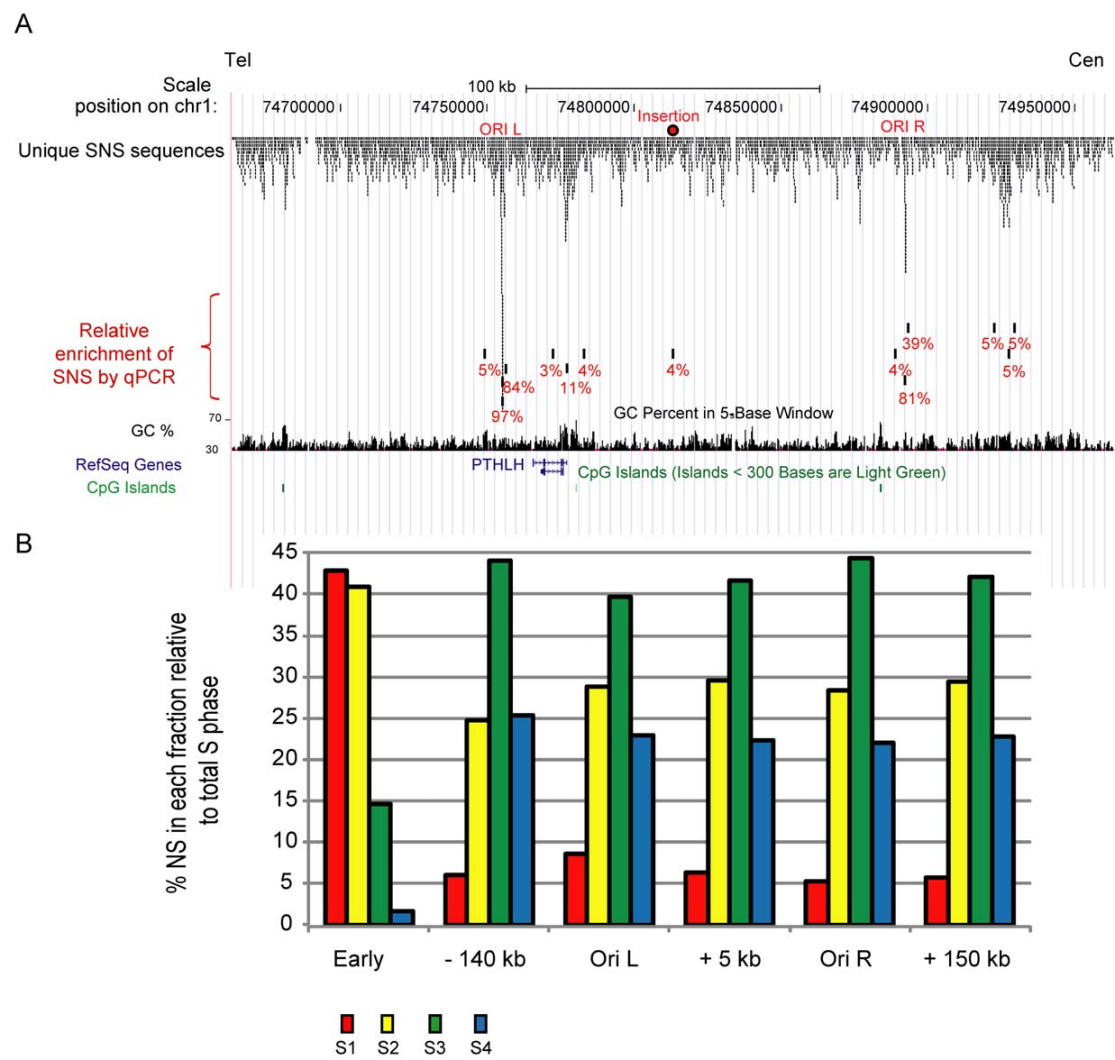
Mapping of replication origins around the selected site of insertion in DT40 cells. (A) UCSC genome browser visualization (May 2006 build) of unique short nascent strand sequence tags aligned to a 300 kb mid-late replicated region of Chromosome 1. Four peaks were identified among which two were validated by qPCR. Tracks showing GC percentage, annotated genes, and CpG islands are below. (B) Precise quantification of replication timing profiles near the site of insertion and along flanking regions. The position 0 corresponds to the site of insertion. The early control is located inside the *β-globin* locus. Cells were BrdU pulse labeled, sorted into four S-phase fractions, and nascent strands were quantified by real-time PCR (S1 in red, S2 in yellow, S3 in green, and S4 in blue).

Our aim was to identify combinations of *cis*-elements capable of imposing early replication timing in a naturally late replicating region. To do so, we employed the efficient homologous recombination capacity of the chicken lymphoid DT40 cell line as a model system [38]. We decided to target an isogenic locus that is replicated in the second part of S-phase and is devoid of replication origins. Firstly, we determined the genome-wide replication timing profiles of DT40 cells following pulse labeling with BrdU and cell sorting into three fractions (early, mid, and late S-phase). BrdU labeled nascent DNA from early and late fractions were immunoprecipitated with anti-BrdU antibody, amplified, differentially labeled, and co-hybridized onto a whole chicken genome oligonucleotide microarray. The log_2_-ratio of the abundance of each genomic probe in the early and late S-phase fractions generates a replication-timing profile that reveals early and late replicated domains (Figure 1A). Exclusion of the mid-replicated fraction increases the intensity of the differences between early and late replicating domains without changing the global shape of the pattern (compare Figure 1A and 1B). We made replicate experiments with nascent strands extracted from cells sorted into early and late-replicating DNA fractions, which were reciprocally labeled (“dye-switch”) prior to hybridization. The mirror image of the timing profiles reflects a high degree of correlation (Figure 1B). We also constructed a whole genome map of replication origins in DT40 cells to allow the identification of origin free regions. We prepared four independent biological samples of short nascent strands (SNS) from 10^8^ cells as described previously [17]. SNS, by contrast to NS, which are synthesized along the whole genome, are only enriched at replication starting points. SNS were pooled and made double stranded by random-priming and ligation. DNA was then fragmented and two different libraries were constructed and subject to high throughput sequencing. A total of 60 million uniquely mapped reads were generated. We arbitrarily selected a mid-late replicating region on chicken chromosome 1 that is devoid of replication origins (Figures 1A and 2). The chromosomal landscape of the chosen integration site in DT40 cells is AT-rich and lacks transcriptionally active genes (Figure 2A). The closest origins, detected by deep sequencing and validated by qPCR, are located 58 kb upstream and 80 kb downstream of the site of insertion (Figure 2A). We analyzed replication timing more precisely by sorting BrdU pulse-labeled cells into four S-phase fractions, from early to late (S1–S4). We quantified nascent strands (NS) across the chromosomal region surrounding the site of integration. The region from 140 kb upstream to 150 kb downstream of the site of integration was found to be mid-late replicating (Figure 2B), in agreement with our genome-wide profiling of replication timing (Figure 1A). In conclusion, we identified and selected a 100 kb intergenic region devoid of replication origins that is replicated in mid-late S-phase. The capacity to specifically target this region by homologous recombination gave us a model system in which we can test the effect of *cis*-regulatory elements on replication dynamics in a very controlled manner.

### The Combination of a Strong Replicator, Transcription, and Histone Acetylation Is Not Sufficient to Impose Early Replication

**Figure 3:**
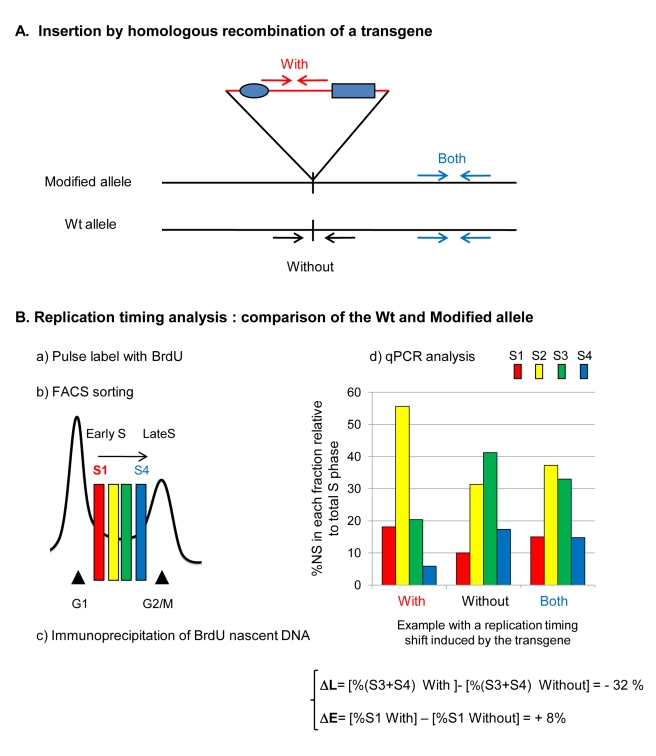
Analysis of replication timing changes after the insertion of *cis*-regulatory elements. (A) Diagram showing the modified allele after insertion of a transgene by homologous recombination and below the wild type allele. Primer pairs used for replication timing analyses are shown and can detect replication timing of the modified allele (With), the wild type allele (Without), or both alleles (Both). (B) After insertion by homologous recombination, selected clones are pulse labeled with BrdU and cell sorted into four fractions from early to late S-phase. BrdU labeled nascent strand DNA is immunoprecipitated and quantified by real-time PCR. The nascent strands produced at the site of integration on the allele containing the transgene (With), the wild type allele (Without), or both alleles (Both) were quantified. Analyses of both alleles should display an average picture of the profiles obtained on each allele, thus providing a validation of the PCR quantification. For each clone, we calculate differences in the timing of replication between the unmodified (Without) and targeted (With) alleles, where ΔL and ΔE describe the differences in late and early S-phase, respectively.

We used homologous recombination in DT40 cells to target transgenes carrying combinations of regulatory elements into one allele at chr1: 74,813,240. We isolated and quantified NS produced at the site of integration on either the allele containing the transgene (With), the wild type allele (Without), or both alleles (Both) during early to late S-phase (Figure 3A and 3B). We present analyses from single independent cell sorts, where each data point is the average of at least two independent PCR quantifications. Analyses of both alleles should display an average picture of the profiles obtained on each allele, thus providing a validation of the PCR quantification (Figure 3B). We have analyzed two independent clones or two cell sorts to confirm the robust reproducibility of the timing profiles shown. Finally, in order to compare precisely the extent of the replication timing shift between constructs, we calculate for each cell line the changes occurring on late fractions (ΔL) and on the earliest fraction (ΔE) as follows: ΔL = [%(S3+S4) With−%(S3+S4) Without] and ΔE = [%S1 With]−[%S1 Without] (Figure 3B). The addition of S2 to S1 would lead to a number equal in absolute value to the sum of change in S3 and S4. We therefore decided to calculate only the change in S1 that provides complementary information on the extent of the replication timing shift. We consider that we have a significant replication timing shift when ΔL≤−10% and ΔE≥+5%.

**Figure 4:**
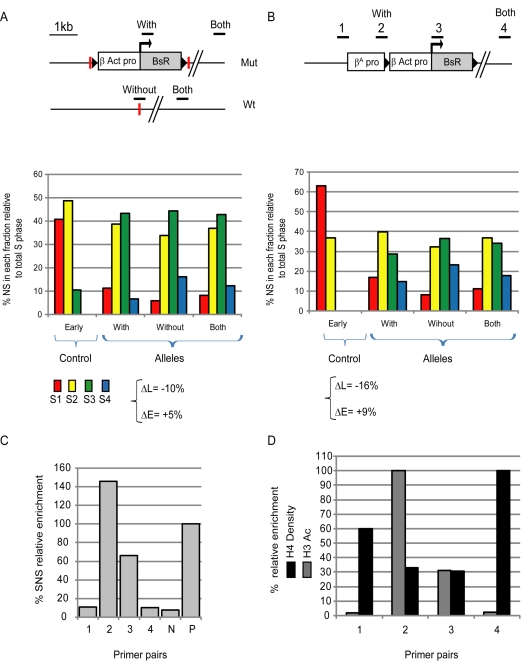
A strong origin linked to a transcriptionally active transgene has a minimal effect on replication timing. (A and B) Replication timing profiles of chromosomal alleles following targeted integration of transgenes into a late replicating locus of DT40 cells described in Figure 2. (A) Analysis of a transgenic line containing a blasticidin resistance gene (grey rectangle) under the control of the *β-actin* promoter (white rectangle) targeted into the site of integration (red vertical bar). The modified (Mut) and wild type (Wt) alleles are represented together with the location of amplicons used for timing analysis. (B) Analysis of a transgenic line containing the same transgene linked to the *β^A^-globin* promoter/replicator. Only the modified allele is shown with the position of amplicons used in QPCR analyses. (A and B) Cells were BrdU pulse labeled, sorted into four S-phase fractions and nascent strands quantified by real-time PCR (S1 in red, S2 in yellow, S3 in green, and S4 in blue). Three different PCR primer sets were used to measure the replication timing at the site of integration on either the transgenic allele (With), the endogenous allele that lacks transgene integration (Without), or both alleles (Both). The endogenous *β-globin* locus was analyzed as an early replicated control. The differences in late or early replication (ΔL and ΔE) at the target site following transgene integration are shown. (C) Quantification of SNS enrichment over the *β^A^-globin*/*β-actin* transgene. Three transgene-specific primer sets were used (1–3, indicated in B); primer set 4 is located 5 kb from the integration site. SNS enrichments are relative to those of the endogenous *ρ-globin* positive control origin (P). A region located 21 kb upstream of the endogenous HS4 insulator that is devoid of origin activity was analyzed as a negative control (N). (D) ChIP analysis of H3K9acK14ac and H4 density along the *β^A^-globin/β-actin* transgene. Histone H3 acetylation levels are relative the *β-actin* promoter (region 2) and H4 density is relative to distal region 4.

In the first targeting experiment, we introduced a blasticidin resistance transgene under the control of the strong chicken *β-actin* promoter. Cells are maintained in the presence of blasticidin and must therefore continue to transcribe the resistance gene. When comparing the allele carrying the blasticidin resistance gene with the wild type allele, we observe a faint shift in replication timing (Figure 4A) (ΔL = −10% and ΔE = +5%). Analysis of both alleles shows an intermediate profile. Therefore, the introduction of an actively transcribed gene has little impact on replication timing at this chromosomal region. We then added the strong *β^A^-globin* origin (the *β^A^-globin* promoter) to this transgene, which results in a more pronounced shift in replication timing (Figure 4B) (ΔL = −16% and ΔE = +9%). We confirmed that the *β^A^-globin* promoter has strong origin activity at this chromosomal site as it is highly enriched in short nascent strands (SNS) (Figure 4C, primer pair 2). We also detected origin activity at the *β-actin* promoter, although lower than that at the *β^A^-globin* promoter (Figure 4C, primer pair 3). This is in agreement with the observation that the blasticidin resistance transgene under the control of the strong chicken *β-actin* promoter induces a faint replication timing shift (Figure 4A). We also performed chromatin immunoprecipitation assays for histone H3 acetylation (H3K9acK14ac) and histone H4 density. We found that the adjacent *β^A^-globin* promoter replicator and *β-actin* promoter are highly enriched for H3 acetylation and are locally depleted of nucleosomes (Figure 4D). Taken together, these results show that transcriptionally active, open chromatin is not sufficient to trigger a significant shift to early replication, despite the proximity of a strong origin. This replication timing shift is similar to the one previously obtained when the human β-globin origin is located near a site of histone acetylation [7] (ΔL = −14% and ΔE = +3%) and therefore suggests that H3 acetylation only acts to fine tune the regulation of replication timing [11].

### Flanking a Strong Origin with Insulator Elements Induces a Substantial Shift Toward Earlier Replication

**Figure 5 pbio-1001277-g005:**
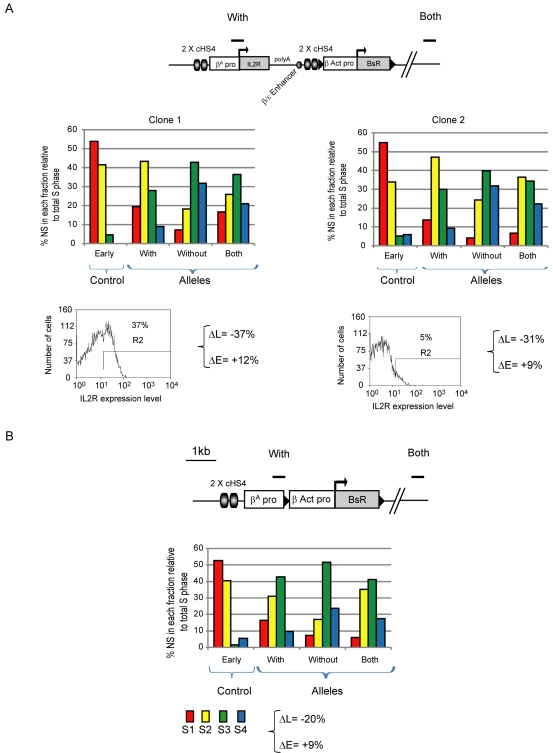
An origin flanked by HS4 insulator elements induces an important early replication shift. Replication timing profiles of DT40 chromosomal alleles following targeted integration of insulator containing transgenes. (A) Analysis of a transgenic line containing an *IL-2R* transgene flanked by two copies of the 275 bp HS4 insulator linked to the *β-actin* transgene. The *IL-2R* cDNA (grey rectangle) and the SV40 polyA signal were linked to the *β^A^-globin* promoter (white rectangle) and the *β/ε* enhancer (grey oval). Flow cytometric analysis of *IL-2R* expression is shown below for the two transgenic lines (clones 1 and 2). The percentage of cells with fluorescence levels higher than control cells (R2) are indicated. (B) Analysis of a transgenic line containing two copies of the 275 bp HS4 insulator upstream of the *β^A^-globin*/*β-actin* transgene described in Figure 4B.

We next tested whether additional *cis*-regulatory elements might cause a more dramatic replication timing shift toward the first half of S-phase. To this aim, we made use of transgenes flanked by HS4 insulator elements, which are protected from chromosomal silencing and also contain the *β^A^-globin* promoter replicator [31],[34],[35]. The HS4 insulator may assist the early firing of a replicator as it recruits active histone modifications, protects against DNA methylation, and can form the boundaries of chromatin domains [32]–[35]. We reasoned that such protected chromatin environment could be important for the formation of an early replicated domain. The *IL-2R* reporter transgene with its erythroid-specific regulatory elements and two flanking copies of the HS4 insulator were integrated with the selection marker gene (Figure 5A). We confirmed by flow cytometric analysis of IL-2R expression that the transgene is weakly transcribed or inactive in the lymphoid DT40 cell lines (Figure 5A). Despite the lack of transcriptional activity from the transgene, this construct induces a substantial shift in replication timing from the second half to the first half of S-phase (ΔL = −37% and ΔE = +12% for clone 1, ΔL = −31% and ΔE = +9% for clone 2). This result suggests that the HS4 insulator contains *cis*-elements that control replication timing.

We next assessed whether the addition of only two copies of the 275 bp core HS4 insulator element next to the *β^A^* replicator was sufficient to increase a shift toward mid-early replication (Figure 5B). We observed only a slight increase in the replication shift compared to the same construct devoid of 2× HS4 (ΔL = −20% and ΔE = +9%) (compare Figures 5B and 4B). This suggests that the HS4 insulator has to flank the *β^A^-globin* promoter replicator in order to control replication timing, although we cannot exclude the contribution of other *cis*-elements found in our construct such as the β/ε enhancer. Altogether these results suggest that HS4 contains an activity that can control replication timing when flanking a replicator and that cooperation with an increasing number of *cis*-regulatory elements intensifies the extent of the replication timing shift.

### USF Elements Can Regulate Replication Timing

**Figure 6 pbio-1001277-g006:**
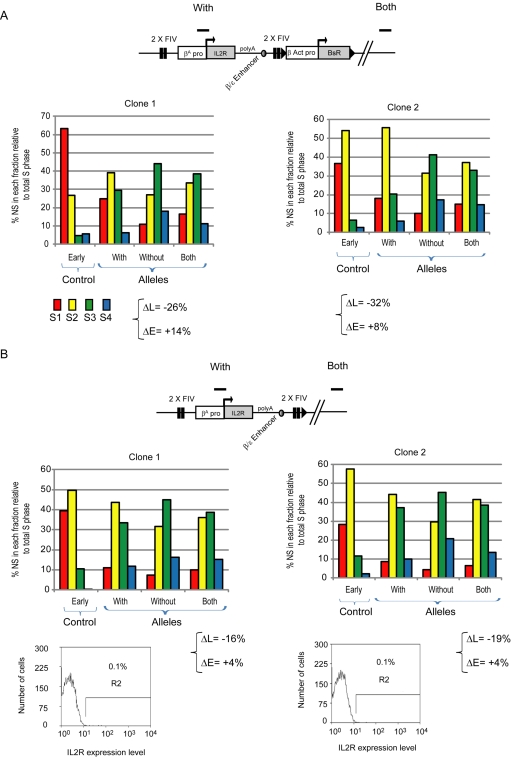
The USF binding site from the HS4 insulator is sufficient to direct early replication. (A and B) Replication timing profiles of chromosomal alleles following targeted transgene integration. The differences in late or early replication (ΔL and ΔE) at the target site following transgene integration are shown. (A) Analyses of two clonal cell lines containing the *IL-2R* transgene flanked by two copies of the FIV USF binding site linked to the *β-actin* transgene. (B) Analyses of two clonal cell lines containing the same construct after recombinase-mediated excision of the *β-actin* transgene. Flow cytometric analysis of *IL-2R* expression is shown below for the two transgenic lines (clones 1 and 2).

We next examined which components of the HS4 element contribute to the control of replication. The HS4 insulator is a composite element containing five binding sites (FI-FV) for three different insulator proteins: CTCF (FII), VEZF1 (FI, FIII, and FV), and USF1/USF2 (FIV). The USF binding site is required for the recruitment of active histone modifications to the HS4 insulator and USF1 interacts with histone modifying enzymes [32]–[34]. Given that histone acetylation was shown to control replication timing in S. *cerevisiae* and mildly influence replication timing in mammals [7],[8], we therefore considered that the USF binding element FIV might be the key regulatory element providing timing information in our system. To address this issue, we assembled a construct where the HS4 insulators were substituted with two copies of 23 bp HS4 fragments containing the USF site FIV (Figure 6A). We analyzed two independent clones and found that this construct imposes a shift to earlier replication timing which is comparable to the one observed using the whole insulator (compare Figure 6A with Figure 5A) (ΔL = −26% and ΔE = +14% for clone 1, ΔL = −32% and ΔE = +8% for clone 2). Therefore, the USF binding site and the activities it recruits is the key component of HS4 that controls replication timing. We also wanted to address whether active transcription was required to provide a significant replication shift. Thus far, every construct that induces a significant shift contains the very efficient *β-actin* promoter and the *blasticidin* resistance gene. Every construct in our targeting experiments was integrated into a DT40 cell line that constitutively expresses an inactivated Cre recombinase fused to the mutated estrogen receptor [39]. The blasticidin resistance marker gene, which is flanked by two mutant loxP sites, is readily excised by transient induction of Cre recombinase activity with 4-hydroxy tamoxifen. We validated the correct excision by PCR and tested replication timing in a new isolated clone. We generated clones in which the blasticidin gene cassette was excised following Cre induction, leaving the *IL-2R* transgene flanked by FIV sites (Figure 6B). Replication timing analyses of two independent clones showed that although the *IL-2R* transgene is transcriptionally inactive, this construct has the capacity to induce a significant replication timing shift (Figure 6B) (ΔL = −16% and ΔE = +4% for clone 1, ΔL = −19% and ΔE = +4% for clone 2). This effect is weaker than the construct linked to the β-actin promoter, consistent with a cooperation between *cis*-elements in the control of the extent of the replication timing shift (compare Figure 6A with Figure 6B).

**Figure 7 pbio-1001277-g007:**
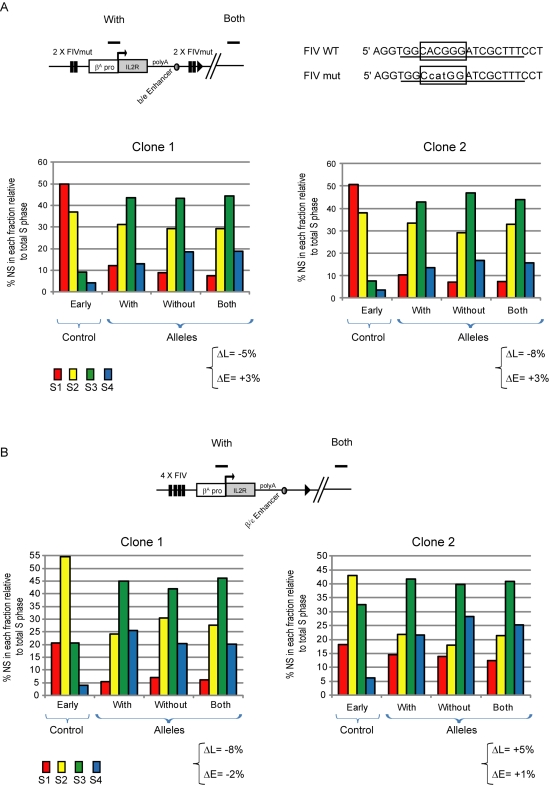
Mutation of USF elements abolishes replication timing control. Replication timing profiles of DT40 chromosomal alleles following targeted integration of insulator containing transgenes. (A) Analysis of two transgenic lines containing the *IL-2R* transgene flanked by two copies of FIV as described in Figure 6B, except that the FIV sites carry E-box mutations known to disrupt USF binding. The sequences of wild type FIV (FIV WT) and mutated FIV (FIV mut) are shown; bases footprinted by USF binding are underlined and the degenerate E-box motif is boxed. (B) Analysis of two transgenic lines containing the *IL-2R* transgene flanked on one side by four copies of FIV. The differences in late or early replication (ΔL and ΔE) at the target site following transgene integration are shown.

The FIV site of the HS4 insulator contains a divergent E-box that is bound by the transcription factors USF1 and USF2 [34]. In order to confirm that USF binding is required for replication timing control, we mutated the E box of FIV (Figure 7A), which abrogates the binding of USF1 and USF2 [34]. The FIV mutation almost abolishes the replication timing shift observed with *IL-2R* transgenes flanked by wild type USF sites (compare Figure 7A with Figure 6B) (ΔL = −5% and ΔE = +3% for clone 1, ΔL = −8% and ΔE = +3% for clone 2). This finding shows that USF binding motifs are critical regulators of replication timing control.

It has been shown previously that this USF binding site is responsible for the recruitment of several histone modifications associated with open chromatin to the endogenous HS4 insulator [34]. We used native chromatin immunoprecipitation (ChIP) assays to monitor histone modifications in DT40 cells following the integration of transgenes flanked by FIV USF sites. Prior to transgene integration, the target locus is highly enriched in the repressive mark H3K27me3, and devoid of active chromatin marks such as acetylated histones H3 and H2A.Z, or methylated H3K4 (Figure S1A). We find that the FIV USF site is sufficient to recruit the active chromatin marks H3K4me2 and acetylated H3, and incorporate acetylated H2A.Z at the transgene (Figure S1C–E). The level of these marks at the single copy transgene are 40%–50% of the levels observed at the endogenous HS4 insulator, consistent with a similar performance of the transgenic and endogenous USF sites in DT40 cells. The presence of the FIV USF sites also results in a depletion of H3K27me3 at the target site (Figure S1F). Mutation of the FIV USF sites considerably reduces the recruitment of active marks and there is no depletion of H3K27me3 at the transgene site (Figure S1C–F). Altogether, these data support the hypothesis that active histone modifications may play a role in advancing replication timing.

### USF Binding Sites Need to Flank the Origin in Order to Drive a Replication Timing Shift

**Figure 8:**
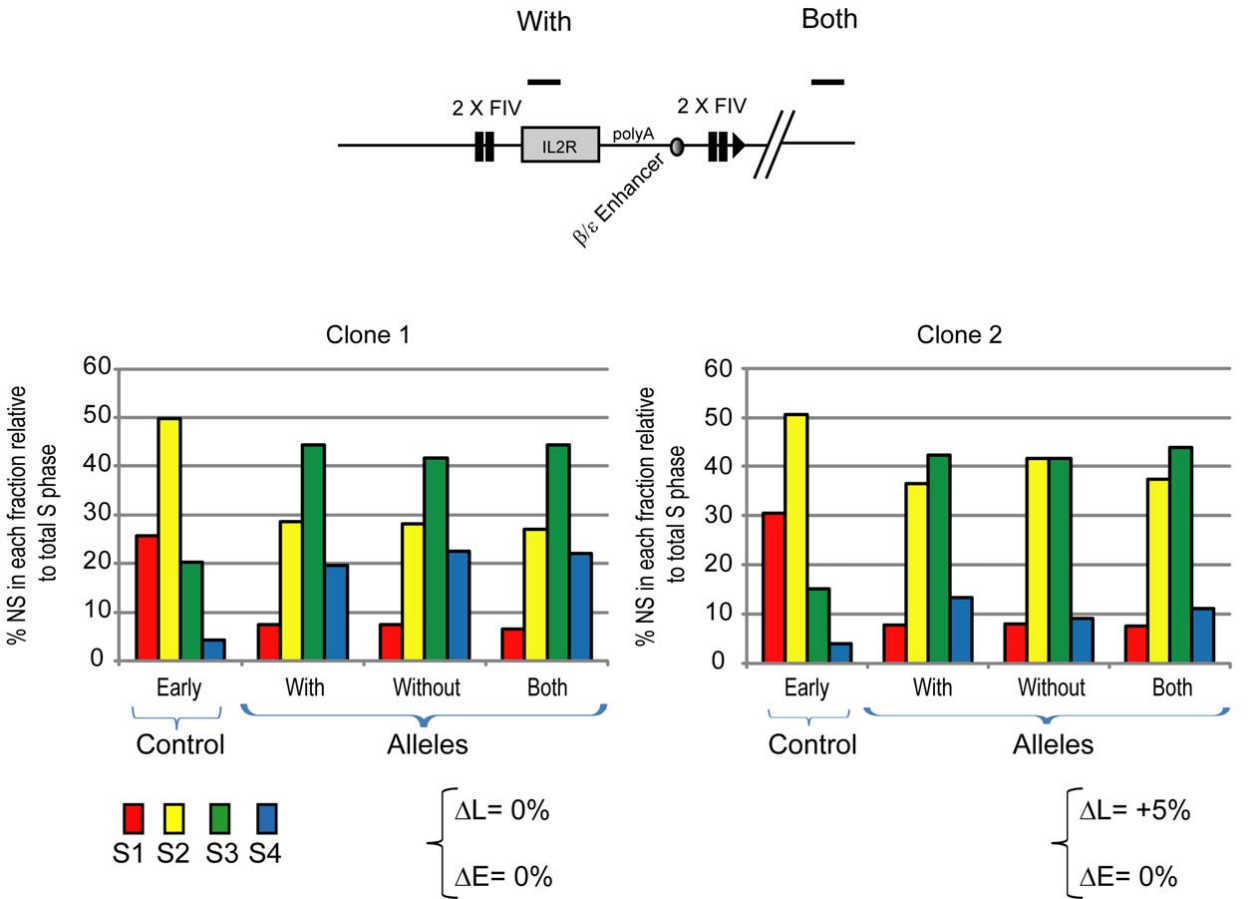
The presence of a replicator is necessary to induce a replication timing shift. Replication timing profiles of DT40 chromosomal alleles following targeted integration of the replicator-deleted transgene. Analysis of two transgenic lines containing the *IL-2R* transgene flanked by two copies of FIV as described in Figure 6B, except that the β^A^ promoter/replicator is deleted. The differences in late or early replication (ΔL and ΔE) at the target site following transgene integration are shown.

Our experiments have shown that the integration of an origin flanked by HS4 or FIV insulator sequences can induce a shift toward early replication. However, the integration of the β^A^ promoter/origin with HS4 insulator sequences only on one side does not significantly increase the replication timing shift (Figure 5B), suggesting that it is necessary to surround an origin with insulator sequences to achieve a significant replication timing shift. In order to confirm this, we analyzed a new construct where four copies of FIV are located just upstream of the β^A^ promoter/origin. This 4× FIV (one side) construct has the same genetic content as that containing pairs of flanking FIV sites (compare Figure 7B with Figure 6B). We generated two independent clones that carry the 4× FIV construct, both of which showed a very weak replication timing shift (ΔL = −8% and ΔE = −2% for clone 1, ΔL = +5% and ΔE = +1% for clone 2). The significant differences observed in the replication timing shift between the 2× FIV (flanking) and 4× FIV (one side) clones demonstrate that the flanking of an origin with sequences that recruit USF proteins is critical for replication timing advancement. Finally, it remains plausible that USF binding sites can induce a replication timing shift in the absence of a proximal replicator. In order to prove that USF binding sites need to cooperate with a functional replicator, we generated two independent clones containing a construct similar to the one described in Figure 6B but deleted for the β^A^ promoter/origin (Figure 8). Both showed no replication timing shift (ΔL = 0% and ΔE = 0% for clone 1, ΔL = +5% and ΔE = 0% for clone 2), thus demonstrating the necessity of strong proximal replicator.

### The *β^A^* Origin Flanked by USF Binding Sites Creates an Independent Early Replicating Domain

**Figure 9:**
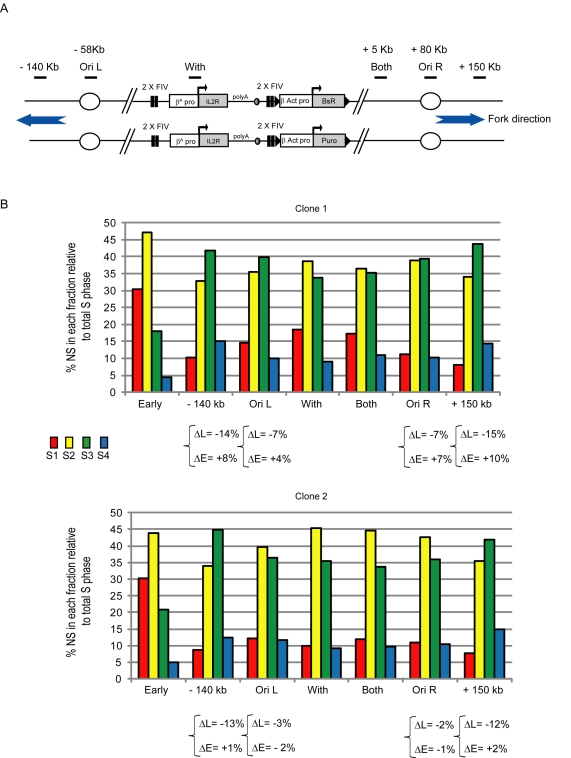
The replication timing shift affects only the replicon located inside the transgene. Replication timing profiles surrounding DT40 chromosomal alleles following transgene integration into both alleles. (A) Schematic representation of the transgenic locus containing the *IL-2R* transgene flanked by two copies of the FIV USF binding site linked to the *β-actin* blasticidin resistance transgene on one allele and puromycin resistance transgene on the other. Primer pairs used are located at the top of the figure, and their positions relative to the site of insertion are indicated. The global replication forks' directions deduced from panel B are shown. (B) Analysis of replication timing at the integration site and over a ∼300 kb region surrounding the transgene integration site in two clonal cell lines. The differences in late or early replication (ΔL and ΔE) at flanking regions compared with the integration site (With) are shown.

We next asked whether the early replication timing we observe at the transgene integration site extends into flanking chromosomal regions. This should depend on the presence of active origins nearby, the capacity of transgenic *cis*-elements to influence the replication timing of remote origins, and the speed of replication forks coming from the transgene. We targeted a DT40 cell line containing the 2× FIV (flanking) construct linked to the blasticidin gene on one allele with a similar construct carrying the puromycin resistance gene (Figure 9A). The targeting of both alleles with a construct that resulted in the largest timing shift at the integration site allows us to study the advancement of replication timing at remote sites. We then analyzed replication timing using primer pairs located inside the transgene (With), near the site of integration (Both), at the flanking endogenous origins (Ori L and Ori R) and distantly from the site of integration (∼150 kb) (Figure 9A).

We determined earlier that the replication timing for 150 kb around the integration site is consistently mid-late replicating in non-transgenic DT40 cells (Figure 2B). Consistent with our earlier analysis of targeting one allele, integration of the 2× FIV transgene construct at both alleles results in a shift to earlier replication at the integration site (Figure 9B). The replication timing patterns for the With and Both primer pairs are indistinguishable, confirming that both alleles are correctly targeted (Figure 9B). Replication timing at the nearest endogenous origins Ori L (−58 kb) and Ori R (+80 kb) is comparable to the integration site in clone 2, but slightly delayed in clone 1. However, the transgene-induced shift to early replication diminishes significantly at positions −140 kb and +150 kb in both clones (Figure 9B). DNA replication in chicken DT40 cells is mostly bi-directional, where the average rate of fork progression is ∼1.5 kb/min [40]. We would anticipate that forks should advance ∼90 kb from the site of replication initiation during the 1 h BrdU pulse labeling. Our observations indicate that only the integrated transgenes contain mid-early firing origins. The advanced replication timing of their chromosomal neighborhood appears to be due to the uni-directional progression of a replication fork initiated at the *β^A^-globin* replicator, showing that replication timing is locally controlled at one individual replicon.

### Insulator Constructs Induce Replication Timing Shifts at Other Chromosomal Loci

**Figure 10:**
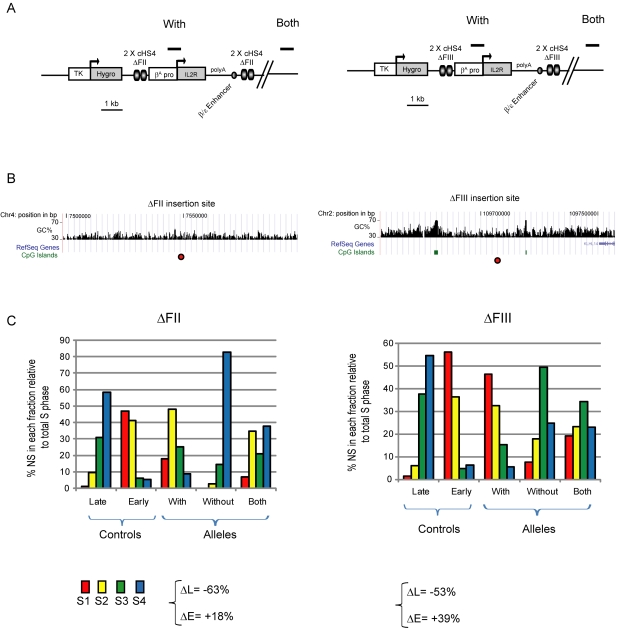
Integration of a transgenic *β^A^* replicator flanked by insulators induces early replication timing at two other chromosomal loci. Replication timing profiles following transgene integration into erythroid 6C2 cells. (A) Schematic representation of the *IL-2R* transgene drawn to scale. The construct is identical to the one described in Figure 5A, except that HS4 carries deletions of the CTCF (ΔFII) or VEZF1 (ΔFIII) binding sites. This construct is linked to the hygromicin antibiotic resistance gene under the control of the HSV-thymidine kinase promoter. (B) The mapped sites of integration (red dots) for the transgenes flanked by ΔFII or ΔFIII insulators are shown. UCSC genome browser views (May 2006 build) of 100 kb regions with the chromosomal position, GC percentage, annotated genes, and CpG islands shown. (C) Replication timing profiles of chromosomal alleles at the mapped sites of transgene integration. Late and early replicated controls correspond to the endogenous *amylase* α 2A and *β-globin* loci, respectively. The differences in late or early replication (ΔL and ΔE) at flanking regions compared with the integration site are shown.

We have shown that the HS4 insulator in cooperation with other *cis*-regulatory elements can induce the early replication of a specific mid-late replicating locus in DT40 cells. Next, we wanted to address whether similar constructs could induce earlier replication at other chromosomal loci and in another cell type. We therefore studied transgenic 6C2 chicken erythroid cell lines that contain exactly the same *IL-2R* reporter transgene cassette flanked by mutant insulator elements [31]. These transgenic cells contain a co-integrated hygromycin resistance gene under the control of the HSV-thymidine kinase promoter. We analyzed cell lines that contain a single copy of the *IL-2R* transgene, flanked by HS4 elements that are devoid of either its enhancer blocking (ΔFII) or barrier (ΔFIII) activities (Figure 10A) [31]. These cell lines have been extensively characterized for transgene expression during prolonged culture, histone and DNA modifications, and insulator protein binding [31],[34],[35]. We cultured early passage cells for 40 d to allow for chromosomal position effect silencing to occur. Using flow cytometry, we confirmed previous findings that *IL-2R* expression remains stable when the transgene is flanked by intact HS4 barrier elements (ΔFII) but succumbs to chromosomal silencing without barrier activity (ΔFIII) (Figure S2B) [31].

We then mapped replication origins across the *IL-2R* transgene by quantifying the abundance of short nascent strands (SNS). We find that the *β^A^-globin* promoter performs as an autonomous replicator as it displays strong replication activity at various exogenous loci. For the two cell lines, SNS enrichment at the transgenic *β^A^-globin* promoter (position 2, Figure S2A and C) is greater or equal to that at the endogenous *ρ-globin* origin of replication (P), and at least 20 times higher than an endogenous negative control locus (N) that is devoid of origin activity (Figure S2C). We also find that, like inside the endogenous locus, transgenic HS4 elements (positions 1 and 4, Figure S2A and C) tend to be enriched in SNS, although never as highly as the *β^A^-globin* promoter.

We mapped the genomic locations and replication timing of the ΔFII and ΔFIII transgene integration sites. The transgene flanked by ΔFII insulators is located within an AT-rich region lacking CpG islands (CGI) and the nearest known gene is over a megabase away (Figure 10B). The integration site of the ΔFII transgene is a naturally late replicating region and insertion of the transcriptionally active transgene induces a dramatic shift of replication timing to mid-early S-phase (Figure 10C and Figure S2D) (ΔL = −63% and ΔE = +18%). The transgene flanked by ΔFIII insulators is also located in an intergenic region, but within a more GC-rich context (Figure 10B). We found that the integration site of the ΔFIII transgene is naturally a mid-late replicating region and insertion of the transgene induces a dramatic shift of replication timing to early S-phase (Figure 10C and Figure S2D) (ΔL = −53% and ΔE = +39%). This replication shift occurs despite the transcriptional silencing and DNA methylation of this transgene (Figure S2B) [35]. We note that the replication timing shifts observed in these two erythroid 6C2 cell lines are greater than those seen with similar constructs in DT40 cells. This may be due to inherent differences between the chromosomal loci studied or may be due to an additional contribution of the β/ε enhancer which is functional only in erythroid cells.

In conclusion, we demonstrate that the insertion of a transgene containing a strong replicator, flanked by HS4 insulators in proximity to a transcribed gene, shifts the timing of two heterologous late replicating loci to early S-phase in erythroid cells. Moreover, neither the enhancer blocking nor barrier activities of the HS4 insulator are required to induce this important shift. We note that despite the loss of barrier activity and transgene silencing, the ΔFIII mutant insulators in the same cell line we studied here remain bound by USF proteins and recruit active histone modifications [34],[35]. The ΔFIII transgene in erythroid cells (Figure 10) therefore functions in the same way as the transgene flanked by FIV USF sites in lymphoid cells (Figure 6A). Taken together, our results show that USF binding sites can induce the early firing of a proximal replicator in an otherwise late replicating chromosomal environment.

## Discussion

The molecular mechanisms that control the replication timing program in metazoan are not clearly understood. To what degree the firing of individual replicons is controlled by the chromosomal environment and whether they can be regulated independently remain open questions. There is growing evidence that *cis*-regulatory elements known to organize higher order chromatin structure can influence DNA replication in addition to the regulation of gene transcription. In this study, we have addressed whether a well-characterized insulator element that is known to organize discrete chromosomal domains can control the replication timing of proximal origins. We have carried out most of our analyses in a DT40 model system, where different combinations of gene regulatory elements are integrated into an isogenic test locus. This allows the direct comparison of their ability to control the initiation and timing of DNA replication.

We found that the chicken *β^A^-globin* promoter origin satisfies the definition of a replicator since it is a strong replication initiation site at different chromosomal loci, and its origin activity does not require transcriptional activity. The timing of replication firing from the *β^A^* origin follows that of its chromatin environment. However, when the *β^A^* replicator is flanked by HS4 insulator elements, it can direct early replication in an otherwise mid-late replicating region. The additional proximity of a transcriptionally active gene can further advance replication from this origin to the first half of S-phase.

A previous study in murine cells used recombinase-mediated cassette exchange to study the ability of various combinations of *cis*-elements to influence the replication timing of a randomly selected locus [41]. It was found that constructs containing a portion of the human *β-globin* locus control region could advance replication timing, but that the introduction of the antibiotic resistance gene used for selection was already sufficient by itself to drive the observed shift. This differs from our study where the insertion of a similar transgene driven the strong *β-actin* promoter results in a minor shift in replication timing. One explanation could be that the site of insertion used in the previous study is more prone to a replication timing shift than the one we have selected. Irrespective of the potential inherent differences between chromosomal environments, we found that constructs which combine a replicator with insulator sequences and a strong promoter can drive dramatic shifts toward early replication at three different chromosomal loci in two different avian cell types.

### The Potential Role of the Chromatin Environment in Regulating Origin Firing

**Figure 11:**
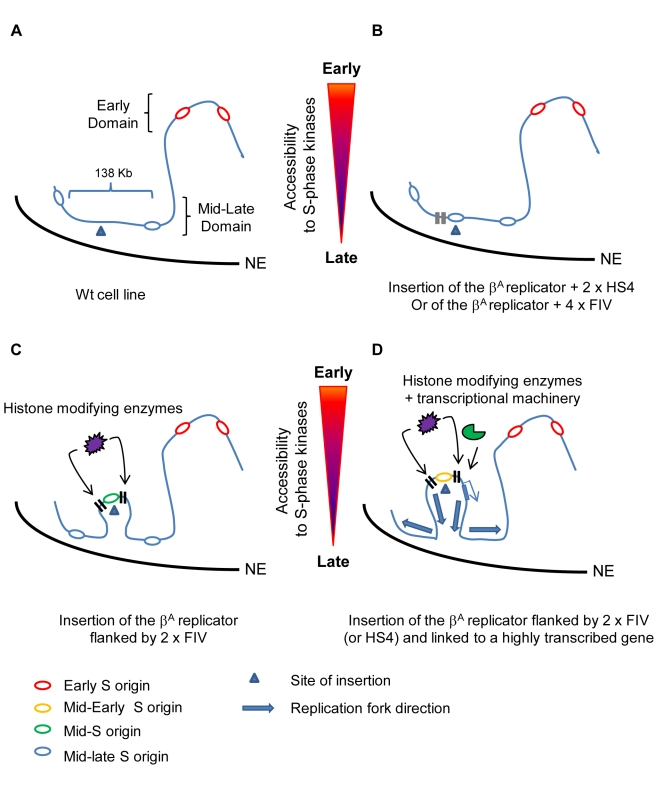
Role of *cis*-regulatory element in controlling replication timing. (A) In wt DT40 cells, the selected site of insertion is located in a mid-late replicating region devoid of strong replication origin. One hypothesis is that this locus is naturally located more toward the nuclear envelope (NE). (B) Insertion of a strong replicator linked to two copies of the HS4 insulator or four binding sites for USF (FIV) does not induce a replication timing shift. The replication timing of the transgenic replicator follows that of its chromosomal environment. (C) Flanking the strong replicator with two copies of FIV induces a significant replication timing shift and recruitment of histone modifying enzymes. This could be accompanied by the formation of a small loop that relocates the replicator more toward the interior of the nucleus. The increased accessibility to S-phase kinases could be either due to local decondensation of the chromatin structure and/or to the nuclear repositioning of the modified chromosomal region. (D) The addition of a strong promoter nearby increases the replication timing shift and leads to the formation of an independent mid-early replicon.

This study identifies the chicken *β-globin* HS4 insulator element as a potential regulator of origin firing. The shift to early replication of the *β^A^* replicator was achieved only when the origin was flanked on both sides by insulator sequences (Figure 11B and C and Table S1). The HS4 elements may be shielding the *β^A^* replicator from processes in its chromosomal environment that suppress early firing. A recent genome-wide study in *Drosophila* showed that H4K16 acetylation (a chromatin modification characteristic of active domains) is more closely correlated with early replication than it is with transcription [6], suggesting that active histone marks such as this might be involved in replication timing control. Our study pinpoints the binding site for USF proteins as the key *cis*-element of the HS4 insulator responsible for the induction of early replication timing control. The USF site is sufficient to direct active H3K4 methylation and histone acetylation, supporting the view that active histone modifications at an origin might favor earlier replication timing (Figure 11C).

In eukaryotic genomes, DNA replication timing and the positioning of chromosomal domains within the nucleus are linked. During early G1, the replication timing program is re-established at the timing decision point (TDP), which is coincident with the repositioning of chromosomal domains within the nucleus [22]. Early replicating domains tend to be positioned toward the center of the nucleus, whereas late replicating domains are typically near the periphery. Furthermore, the Hi-C method of studying chromosomal interactions reveals a close correlation between replication timing and the spatial separation of chromosomal compartments [42]. It is possible that the histone modifications or other activities recruited to USF binding sites may influence their 3-dimensional nuclear localization, which in turn controls the replication timing of origins within their proximity (Figure 11C). Moreover, the observation that replication origins located 58 kb upstream and 80 kb downstream do not fire at the same time as the transgene origin suggests that the advanced timing is restricted to a single replicon and does not involve broader reorganization of a large chromosomal region (Figure 11D). It would be of great interest to study the nuclear positioning of late replicating regions before and after the targeting of *cis*-regulatory elements that control replication timing. It would be important to determine whether changes in replication timing are always accompanied by sub-nuclear repositioning.

Our study shows a correlation between the extent of the replication timing shift and the number of *cis*-regulatory elements surrounding the *β^A^* replicator (Figure 11C and D). In agreement with previous studies, the presence of a replicator is necessary to induce a replication timing shift [43] and the proximity of a transcriptionally active gene can further advance replication timing [44]. This suggests that these elements act to increase the probability of the molecular events that govern the early firing of a proximal replication origin. We speculate that USF elements promote chromatin opening and/or position proximal origins into nuclear compartments that increase the accessibility of pre-RCs to replication factors needed for origin firing during S-phase [45]–[47]. It is also possible that the recruitment and activity of factors like DDK (Dfp1/Dbf4-dependent kinase Cdc7) and/or CDK (Cyclin dependent kinase) may be regulated by USF-directed post-translation modifications of either the histones and/or replication factors.

### The Relationship Between Replication Timing and Gene Expression

Recent genome-wide studies in both *Drosophila* and mouse find that replication timing and gene activity are not tightly coupled. Genes replicated in the first half of S-phase have an equal probability of being active and 10%–20% of late replicating genes are expressed [5],[6]. In agreement with these studies, we also find that transcription is neither necessary nor sufficient for the induction of early replication (Figures 4A and 6B) and moreover that maintaining a transgene in an early replicating domain is not sufficient to prevent its silencing (Figure S2B and D, ΔFIII transgene). However, analyses of a large subset of genes highlight classes of genes showing a link between their expression and their timing of replication, implying a potential role for replication timing in both transcriptional activation and repression. In *Drosophila*, genes related to wing development are replicated earlier in a cell line derived from imaginal discs than in embryonic cells even though these genes are transcriptionally inactive in both cell types [6]. This might reflect an open chromatin state poised for subsequent activation. In mouse, analyses of embryonic stem cell differentiation into neuronal cells revealed that high and low CpG-density promoters showed distinct behaviors upon switching to a late-replicating environment: only CpG-poor promoters showed a higher tendency toward transcriptional down-regulation [5]. Overall, these results favor a model in which only a specific class of genes is affected by replication timing. Furthermore, a reporter plasmid micro-injected into early or late S-phase mammalian nuclei assembled into active hyper-acetylated and inactive hypo-acetylated plasmids, respectively [48]. Taken together, these studies indicate that c*is*-regulatory elements involved in the control of replication timing may also help to organize chromosomal domains that assist the regulation of gene expression. This study shows that USF binding sites can regulate the formation of early replication domains in avian cells. The ubiquitous expression and high sequence conservation of USF transcription factors suggest that this regulation would occur in most vertebrate cell types [34],[49]. This newly proposed function of USF might be an important means to establish chromosomal domains favoring gene expression along vertebrate genomes.

### Defining the Basic Unit of Early Replicated Domains

The mammalian and chicken genomes are partitioned into isochores with different GC content and gene density [50], and isochores that are high in both GC and genes tend to be replicated early in S-phase. In this study, we show that combining several GC-rich *cis*-elements leads to the formation of an early domain of DNA replication in naturally late or mid-late replicating regions. This minimal combination, although artificial, contains only one GC-rich replicator flanked by two copies of a USF binding site, which is enhanced when linked to a constitutive CpG island (CGI) promoter. These elements are bound by transcription factors that are broadly expressed and are known to be part of many transcriptional regulatory elements. GC-rich isochores have a high probability of containing an efficient combination of strong origins (mostly found near CGI) [12],[51], *cis*-regulatory elements bound by USF, and transcriptionally active genes every 50–100 kb. This combination of elements that we find to be capable of determining an early independent replicon could be the basic unit of most early replicated domains. In agreement with this model, we have found that human USF1 is mostly bound within early replicated regions that are also dense in replication origins (Picard F, Cadoret J-C, Audit B; Arnéodo A, and Prioleau M-N, unpublished data).

## Materials and Methods

### Isolation of Short Nascent Strands (SNS) and Deep Sequencing Analysis

Short nascent strands were purified as described previously [17]. The quality of the preparation was tested by real-time quantitative PCR using primer pairs corresponding to the detection of the endogenous 3′ *ρ-globin* origin (positive control, set arbitrarily at 100%) and to a region devoid of replication origin located 21 kb upstream of HS4 (negative control). The endogenous β^A^-origin has a relative enrichment of ∼130% in most preparations.

For the genome-wide mapping of origins, four preparations of SNS were made independently from 10^8^ cells and then pooled. SNS were made double stranded by random priming and ligation. Two libraries were constructed using illumina protocols, and four deep sequencing were performed using the GA-IIx sequencer (Illumina) generating 76 bp length reads. TheSOAP v2 software was used to map reads to reference chicken genome (UCSC, Gallus gallus Build May 2006) with the r:0, I:30, and v:5 command-line. The enrichment of short nascent strand sequencing reads was detected using the Sole search program with the following parameters: Ref genome = generic, permit = 5, fragment = 200, alpha value = 0.001, and FDR = 1.10–4 [52]. Detected peaks were tested on another SNS preparation by qPCR; out of four peaks identified along the region and shown in Figure 2A, only two had a significant enrichment.

### Replication Timing Analyses

Timing analyses were made as previously described [17] except that S-phase was divided into four fractions from early to late S-phase named S1 to S4 for qPCR analyses and into two or three fractions for DNA microarrays studies. For DNA microarrays, in order to obtain sufficient DNA, immunoprecipitated nascent strands were amplified by whole-genome amplification (WGA) (Sigma). After amplification, early and late nascent strands were labeled with Cy3 and Cy5 ULS molecules (Genomic DNA labeling Kit, Agilent) as recommended by the manufacturer. The hybridization was performed according to the manufacturer instructions on 4× 180K Chicken microarrays (Chicken Genome CGH Microarray 4× 180K, custom microarray design, genome reference Gallus gallus V3 May 2006) that cover the whole genome with one probe every 5.6 Kb. Microarrays were scanned with an Agilent's High-Resolution C Scanner using a resolution of 2 µm and the autofocus option. Feature extraction was done with Feature Extraction 9.1 software (Agilent technologies). For each experiment, the raw data sets were automatically normalized by the Feature extraction software. Analysis was performed with Agilent Genomic Workbench 5.0 software. The log_2_-ratio timing profiles were smoothed using the Moving Average option of the Agilent Genomic Workbench 5.0 software with the linear algorithm and 200 kb windows.

### Flow Cytometry

#### IL-2R expression

Cells growing in exponential phase were harvested and washed once in PBS. Then, 5×10^5^ cells were washed in PBS plus 0.1% BSA pelleted and incubated for 30 min on ice in 10 µl of PE-Cy5 conjugated Mouse Anti-Human CD25 (IL-2R) (BD Pharmingen, ref:555433). Cells were then washed twice in PBS plus 0.1% BSA, resuspended in 500 µl of the same buffer, and analyzed by flow cytometry. Non-transfected cells were used to determine auto-fluorescence levels in the absence of IL-2R expression.

#### Timing analyses

Exponentially growing DT40 wild type cells were washed twice with 1× PBS, fixed in 75% ethanol, and stored at −20°C overnight. Fixed cells were re-suspended in 1× PBS with propidium iodide (PI) (final concentration of 50 µg/ml) and RNAse (0.5 mg/ml) and incubated for 30 min at room temperature. Cells were sorted by using INFLUX 500 (Cytopeia purchased by BD Biosciences) cell sorter. 50,000 cells were sorted in each fraction of S1, S2, S3, and S4 based on the nuclear content. To check the quality of post-sorted cells (Figure S3), the post-sort and pre-sort cells were stained with PI (40 µg/ml) and incubated for 30 min at room temperature before analyzing on the Cyan ADP LX (DakoCytomation acquired by Beckman Coulter) analyzer.

### Inverse PCR

500 ng of genomic DNA extracted from different 6C2 cell lines was digested with *Alu*I, *Hae*III, or *Hpa*II restriction enzymes. 100 ng of digested DNA was used for ligation in a 10 µl volume in order to favor intra-molecular events. 3 µl of the ligation was used for nested PCR with two primer pairs located at the border of the transgene. Specific products were cloned using the TopoBlunt cloning kit (Invitrogen), and recombinant plasmids were sequenced. Sequences were aligned to the chicken genome using the UCSC genome browser (http://genome.ucsc.edu/cgi-bin/hgBlat) and the correct mapping of insertion sites were validated by the amplification of a fragment covering the end of the inserted plasmid and the chicken genomic sequence identified.

### Karyotype Analysis

For karyotype analysis, metaphase chromosome spreads were prepared as previously described [53]. Briefly, ∼10^7^ exponentially growing DT40 cells were treated with 0.1 µg/ml KaryoMAX Colcemid Solution (GIBCO) for 2 h. Harvested cells were treated with 1 ml of 0.9% sodium citrate for 20 min at room temperature. Cells were fixed by washing with 5 ml of freshly prepared 3∶1 mixture of methanol/acetic acid and incubating in the same solution for 20 min at room temperature. Fixed cells were finally dissolved in the 200 µl of methanol/acetic acid and dropped onto ice-cold glass slides pre-wet with 50% ethanol from about 50 cm high. Slides were air dried and applied glycerol with DAPI (1 µg/ml).

### Cell Culture and Transfection

6C2 cells were maintained in α-MEM containing 10% FBS, 2% chicken serum, 1 mM Hepes, 0.05 mM β-mercaptoethanol, and penicillin plus streptomycin at 37°C in 5% CO_2_. DT40 cells were grown in RPMI containing 10% FBS, 1% chicken serum, 2 mM L-glutamin, 0.1 mM β-mercaptoethanol, and penicillin plus streptomycin at 37°C in 5% CO_2_. The karyotype of the wild type DT40 cell line used for every construct was verified and displayed a typical DT40 karyotype nearly diploid with a trisomy of chromosome 2 (Figure S4A). For electroporation 10^7^ exponentially growing cells were resuspended into 800 µl of DT40 medium. The cell suspension was transferred to an electroporation cuvette with 35 µg of the linearized plasmid DNA (final concentration 1 µg/µl) and maintained for 10 min at 4°C. Electroporation was made using a Biorad electroporator set at 25 µF and 750 V. After a further 10-min incubation on ice, cells were transferred to a plate containing 10 ml of DT40 medium. The following day, cells were diluted six times in DT40 medium containing blasticidin at a final concentration of 20 µg/ml. 200 µl of cell suspension were distributed into three 96-well flat-bottom microtiter plates. Plates were left for about 7 to 10 d in the incubator without changing the medium. Isolated colonies were then progressively transferred to larger plates until they reached a 10 ml volume. Genomic DNA was extracted from 5 ml of cultured cells with the DNeasy Blood & Tissue kit (Qiagen), and 100 ng of genomic DNA was analyzed by PCR with a primer pair containing one oligonucleotide inside the construct and one just upstream of the arm used for homologous recombination. At least two positive clones were randomly selected and amplified for further studies. A qPCR on 4 ng of genomic DNA of each selected clone with one primer pair inside the transgene (With), one overlapping the site of insertion (Without) and one near the site of insertion (Both), was performed in parallel with genomic DNA extracted from wild type DT40 cells to confirm that each clone only contains one copy of the transgene and that the transgene has been correctly inserted at the target site following homologous recombination (Figure S4B). For excision of floxed cassettes, cells were cultured for 2 d in chicken medium containing 0.01 mM hydroxyl-Tamoxifen. After serial dilutions of 50, 100, and 300 viable cells per 10 ml, 200 µl of the cell suspension were distributed into three 96-well flat-bottom microtiter plates. DNA from isolated colonies were analyzed by PCR with two primer pairs, one containing one oligonucleotide inside the blasticidin gene and one in the 3′ arm (negative control) and one positive PCR with one oligonucleotide inside the construct and one in the 3′ arm. At least two positive clones were randomly selected and amplified for replication studies.

### Screening for Targeted Integration by PCR

As previously described, primers were designed such that one primer is located in the test construct (β^A^ origin, 5′ GTGCAGCATCAGTGGATAAAGT 3′) and one primer is just upstream of the 5′ arm used in the construct (5′ TCTGCCTTCTCCCTGATAACG 3′) (Figure S5). Thus only the genomic DNA from clones integrated by homologous recombination will be amplified by PCR. The specificity of the PCR products was analyzed by *Eco*RI digestion (Figure S5). To screen cell lines after excision of the Blasticidin cassette, PCR was performed with forward primer in the *IL-2R* gene (5′ CAAAGCCATGGCCTACAAGG 3′) and reverse primer in the 3′ targeting arm (5′ TCATTGTTCTCCAGGCTGTACTC 3′) (Figure S5). Only genomic DNA from cell lines excised for the Blasticidin is amplified by PCR. To screen for the cell lines with homologous recombination on both alleles, PCR was performed with primers located on each side of the integration point (5′ GTGCAGCATCAGTGGATAAAGT 3′ and 5′ GGCCTGAACACTGTGTCAAT 3′) such that the double insertion lines do not produce the corresponding PCR product. To perform all the above stated PCR, we used the Herculase II Fusion DNA Polymerase PCR system (Stratagene) with the condition as follows, the initial denaturation at 95°C for 2 min, and 35 cycles of 95°C for 30 s, 57°C for 30 s, and 72°C for 2 min and a final extension of 72°C for 5 min. PCR products were analyzed on 1% agarose gel (Figure S5).

### Plasmid Constructions

Homologous recombination in DT40 cells was done with plasmids constructed with the multisite gateway pro kit (Invitrogen). Entry clones were constructed by PCR amplification with the Herculase II fusion DNA polymerase (Stratagene) with primers flanked by appropriate attB sites. 5′ and 3′ target arms were amplified from DT40 genomic DNA with primer pairs (5′ arm forward: AGTTTCAGCTGTAAGCCTACA; 5′ arm reverse: CTCTTGTGAATACCTGCTGTC) and (3′ arm forward: CGACTCAACTCTGATGCATTGA; 3′ arm reverse: GGGAAGCAATCTGAATCAGAT) and give 2,058 bp and 2,034 bp amplicons, respectively. The blasticidin resistance gene under the control of the β-actin promoter and flanked by loxP sites was amplified from the pLoxBsr vector with the primer pair (forward: GTCGACGGTATCGATAAGCT and reverse: CGACGGCCAGTGAATTGT) [39]. PCR amplified fragments were used for BP recombination reaction; each final entry vector was verified by sequencing. For every construct the same three entry vectors 5′ arm, Blasticidin resistance, and 3′ arm were used, respectively, as fragments 1, 3, and 4 for a MutliSite Gateway pro LR 4 fragments recombination reaction. The last entry vectors (fragment 2) were constructed by PCR amplifications with the Herculase II Fusion DNA Polymerase (Stratagene) of the construct designed in Gary Felsenfeld's laboratory for testing barrier activity with appropriate oligonucleotides. For fragments containing FIV and FIVmut binding sites, a 3 kb DNA fragment containing βA promoter, the IL2R gene linked to the β/ε enhancer, was amplified with two primers containing two copies of the chicken HS4 insulator FIV region at their 5′ end: 2XFIV5′-F: GGGGACAACTTTGTATACAAAAGTTGAGGTGG**C**ACGGGATCGCTTTCCTAGGTGGCACGGGATCGCTTTCCTCTGCCCACACCCTCCTG 3′; and 2XFIV-R: 5′GGGGACAACTTTGTATAGAAAAGTTGGGTGAGGAAAGCGATCCCGTGCCACCTAGGAAAGCGATCCCGTGCCACCTGATGATCCGTCATCCAGACATG3′; 2XFIV mut5′-F: 5′GGGGACAACTTTGTATACAAAAGTTGAGGTGGCcatGGATCGCTTTCCTAGGTGGCcatGGATCGCTTTCCTCTGCCCACACCCTCCTG3′; 2XFIVmut3′-R: 5′GGGGACAACTTTGTATAGAAAAGTTGGGTGAGGAAAGCGATCCatgGCCACCTAGGAAAGCGATCCatgGCCACCTGATGATCCGTCATCCAGACATG3′.

To generate the 4× FIV (one side) construct, we used PCR amplification of the 2× FIV (flanking) vector. We designed the forward primer such that it anneals to the 5′ 2× FIV region and carries two extra FIV at its 5′ end. Whereas the reverse primer anneals upstream of the 3′ 2XFIV, the PCR product was sub-cloned into a TOPO-TA vector. Selection of a correct clone was made after checking the presence of proper 4× FIV by sequencing and then put into an MutliSite Gateway entry vector.

Oligo sequences used for amplification of 4× FIV: 4× FIV_F: 5′GGGGACAACTTTGTATACAAAAGTTGAGGTGGCACGGGATCGCTTTCCTAGGTGGCACGGGATCGCTTTCCTAAGTTGAGGTGGCACGGG 3′; 4× FIV_R: 5′GCCGCGAGCTGCGGGGACAACTTTGTATAGAAAAGTTGGGTGGATGATCCGTCATCCAGACATGATAAGATACATTGATG3′.

Each entry vector was controlled by sequencing. After LR reaction, final vectors were verified by restriction enzyme digestion with several restriction enzymes. For electroporation, final vectors were linearized by *Sca*I.

The *IL-2R* construct deleted for the origin and flanked by 2XFIV was made by a PCR method. The 1.6 kb *IL-2R* fragment was amplified by forward and reverse primers containing 2XFIV site along with AttB site to use in Gateway cloning. We used Herculase II Fusion DNA Polymerase PCR system (Stratagene) for the amplification with the following conditions: the initial denaturation at 95°C for 2 min, and 35 cycles of 95°C for 30 s, 57°C for 30 s, and 72°C for 3 min and a final extension of 72°C for 5 min. At first, the PCR product was cloned with Gateway BP Clonase system (Invitrogen) to generate the entry vector. The positive *IL-2R* entry vectors were checked for no mutations by sequencing. The final targeting vector was prepared by a four fragment multisite Gateway system (Invitrogen): Forward: 5′GGGGACAACTTTGTATACAAAAGTTGAGGTGGCACGGGATCGCTTTCCTAGGTGGCACGGGATCGCTTTCCTCAAAGCCATGGCCTACAAGG 3′; Reverse: 5′GGGGACAACTTTGTATAGAAAAGTTGGGTGAGGAAAGCGATCCCGTGCCACCTAGGAAAGCGATCCCGTGCCACCTGATGATCCGTCATCCAGACATG 3′.

### Chromatin Immunoprecipitation

Crosslinking ChIP was performed as described previously [13] with anti-diacetylated K9 and K14 histone H3 and anti histone H4 pan antibodies (Millipore, ref# 06-599 and 05-858). Low salt native ChIP was performed on DT40 cell nucleosomes. Nuclei were prepared and treated with lysis buffer (10 mM NaCl, 3 mM MgCl2, 0.4% NP-40, and 10 mM Tris pH 7.5) for MNase (Sigma) digestion in the presence of 1 mM CaCl_2_. The MNase concentration required to yield mostly di- and tri-nucleosomes was firstly determined. For ChIP experiments, three equal aliquots of nuclei were incubated with ½×, 1×, and 2× MNase at 37°C for 17 min to obtain representative di- and tri-nucleosomes. Digestion was stopped with 10 mM EDTA. Soluble chromatin was collected by centrifugation at 2,500 g for 5 min. The three supernatants were combined (S1). The remaining pellets were combined and resuspended in lysis buffer supplemented with 10 mM EDTA and left on ice for 15 min. Chromatin was released by passing through 20 then 25 gauge needles, and collected by centrifugation at 10,000 g for 10 min. The supernatant (S2) was combined with S1 for sucrose gradient fractionation. ∼1.5 mg of S1–S2 chromatin was fractionated on 13.5 ml 5%–25% linear sucrose gradients (Biocomp gradient master) in a SW40Ti rotor at 31,000 rpm for 14 h at 4°C. Fractions containing di- and tri-nucleosomes were pooled and fixed with 0.1% formaldehyde at room temperature for 10 min. The crosslinking reaction was stopped with 0.125 M glycine. Nucleosomes were exchanged into N-ChIP buffer (50 mM NaCl, 5 mM EDTA, 10 mM Tris pH 7.5) using P-6DG Bio-Gel (BioRad).

50 µg of nucleosomes were pre-cleared with 5 µg of non-immune IgG and 100 µl (50% slurry in N-ChIP buffer) of protein A/G agarose at 4°C for 3 h. 10 µg of specific antibody were incubated with pre-cleared chromatin at 4°C with agitation overnight. H3K4me2 (07-030), H3K27me3 (07-449), and H3K9acK14ac (06-599) antibodies and H2A.ZK4acK7acK11ac (ab18262) antibodies were obtained from Millipore and Abcam. Binding of protein A/G agarose was carried out at 4°C for 2 h. Immunoprecipitated chromatin was collected and washed 5 times with 1 ml N-ChIP wash buffer (150 mM NaCl, 0.2 mM EDTA, 0.1% Tween-20, and 20 mM Tris pH 7.4). Chromatin was eluted with N-ChIP buffer supplemented with 1% SDS followed by 0.5% SDS. Eluates were digested with Proteinase K at 45°C for 2 h and DNA extracted by phenol/chloroform and precipitated for qPCR analysis.

### Quantification of DNA

Quantitative real-time PCR was performed by using the LightCycler 2.0 Roche detection system with an Absolute QPCR-SYBR Green mix (ABgene). For each reaction, amplification of the purified short nascent strands, BrdUTP-labeled nascent strands, and ChIP DNA were performed in duplicate. Four 4-fold dilutions of total genomic DNA and a reaction mixture without template DNA were used as controls. Subsequent to amplification, a melting curve analysis was performed to analyze the products and to control the specificity of the reaction. The second derivative maximum method was used to quantify sequences, as described in the LightCycler Software. Primer pairs used are listed in Table S2. For short nascent strands analyses, primer pairs overlapping a region with an origin (P) and a primer in a region devoid of origin (N) were used as controls in each reaction. For timing analyses, the abundance of mitochondrial DNA in each fraction was measured by using a specific primer pair (MIT) alongside late and early controls and studied regions. Primer pairs overlapping the site of insertion and next to the site of insertion were used to detect the timing of the wild type allele and both alleles, respectively. A primer pair specific to the transgene was used to analyze the timing of the allele containing the transgene.

## Supporting Information

Figure S1 Transgenic USF binding sites recruit active histone modifications. Native chromatin immunoprecipitation analysis of histone modifications at DT40 loci following transgene integration. (A and B) Histone modification enrichments at the unmodified target allele (A) compared to the endogenous HS4 insulator element (B) as a reference (relative to input DNA). (C–F) Histone modifications in cells containing *IL-2R* transgenes that are flanked by wild type (blue bars) or mutant (red bars) FIV USF sites (as described in Figures 6B and 7A, respectively). The levels of H3K4me2, H3K9acK14ac, H2A.ZK4acK7acK11ac, and H3K27me3 relative to the unmodified target allele are shown in panels C to F, respectively. The levels of each modification at the transgenic FIV sites are shown, compared with those at the endogenous HS4 element and the condensed chromatin region upstream of the *β-globin* locus.
(TIF)

Figure S2 Origin activity of the *β^A^-globin* promoter is independent of its transcriptional activity, chromatin structure, and chromosomal position. (A) Schematic representation of the *IL-2R* transgene drawn to scale with amplicons used for SNS quantification shown below. (B) Flow cytometric analysis of *IL-2R* expression in 6C2 cell lines carrying stably integrated IL-2R transgenes flanked by HS4 insulators that carry deletions of the CTCF (ΔFII) or VEZF1 (ΔFIII) binding sites. Cells were cultured for 40 d following removal of selection. Non-transfected 6C2 cells were used to determine the fluorescence of *IL-2R* negative cells. Cells expressing *IL-2R* have fluorescence levels indicated by the horizontal bars denoted R2. (C) Quantification of short nascent strands by real-time PCR. Enrichments of transgene amplicons were adjusted to enrichments from the endogenous *ρ-globin* positive control origin (P) for each cell line. A negative control region (N) located 21 kb upstream of the endogenous HS4 insulator that is devoid of origin activity was also analyzed. (D) Cells were BrdU pulse labeled, sorted into three S-phase fractions, and nascent strands quantified by real-time PCR (S1 in red, S2 in yellow, S3 in green). Two different PCR primer sets were used to measure the replication timing at the site of integration on either the transgenic allele (With) or the endogenous allele that lacks transgene integration (Without). Late and early replicated controls correspond to the endogenous *amylase* α 2A and *β-globin* loci, respectively.
(EPS)

Figure S3 Analysis of the quality of the post-sorted fractions at the cellular level. The pre-sort (top) and post-sort cells (bottom, S1 to S4) were stained with PI and analyzed by flow cytometry.
(TIF)

Figure S4 Karyotype analysis of the wild-type DT40 cell line used for every construct and validation by qPCR analysis of single targeted insertion of the transgene. (A) The figure shows two examples of a chromosomal spread. Fifteen chromosomal spreads were used to score the number of total chromosomes and especially the macrochromosomes, which are clearly distinguishable from each other. Consistent with the previous reports, we have scored 80 total number of chromosomes in DT40 cells used in this study [53]. A typical DT40 karyotype comprising the clearly distinct 11 autosomal macrochromosomes and one Z sex-chromosome (2Gga-1, 3Gga-2, 2Gga-3, 2Gga-4, 2Gga-5, 1Gga-Z) has been observed. (B) The table shows qPCR results obtained with genomic DNA extracted from clones selected for experiments shown in Figures 4–8 (description of the clones is shown in Table S1). For each clone, 4 ng of genomic DNA was amplified with the three primer pairs (With, Without, and Both) described in Figure 3. Amplification was also performed in parallel on wild type DT40 genomic DNA with primer pairs Without and Both. The crossing point (cp) was obtained by using the second derivative maximum method described in the LightCycler Software (Roche). Each primer pair has a PCR efficiency around 95%; therefore, the difference in the crossing point (cp) between one and two copies should approximate one cycle. Analysis of transgene copy number shows that for most of the clones, a difference of one cycle is observed between the primer pair With (one copy) and Both (two copies). Three clones have a difference ≥1.5 due to slight variations in the efficiency of this primer pair that we have repeatedly observed. In any case this difference does not account for multiple copy insertion. When comparing the number of copies of the region containing the site of integration with a region located nearby, we also observed a difference of one cycle in every clone in contrast to the wild type DT40 cell line. This result confirms both the fact that only two copies of this genomic region are present in the cell line and that targeted insertion has occurred on one allele.
(TIF)

Figure S5 PCR validation of clones selected for homologous recombination. (A) Schematic diagram showing genomic region with a site-specific integrated construct. 5′ and 3′ arms of the targeted vector are shown as black boxes. A construct with *β^A^* origin (blue) linked to the *IL-2R* transgene (green) and Blasticidin (BsR) gene cassette (orange) flanked by loxP sites (yellow triangles) is shown. Arrows represent primers used for the analysis of integration of constructs by homologous recombination and correct excision of BsR. (B–D) Agarose gels showing PCR products. (B) PCR products amplified with primer #1 and #2 for testing the correctly integrated cell lines of β^A^+BsR (Lane 1), 2xIns+BsR (Lane 2), (2xIns+β^A^)+BsR (Lane 3), 2xFIV+BsR (Lanes 4,5), 2xFIV (Lanes 6,7), 2xFIVmut (Lanes 8,9), 4xFIV-one side (Lanes 10,11), double insertion lines with 2xFIV+BsR and 2xFIV+Puro (Lanes 12,13), and DT40 wild type as negative control (Lane 14). PCRs from genomic DNA extracted from correctly integrated cell lines should give a 2.3 kb product, except those containing 2xIns (Lanes 2 and 3), which should give a 3 kb product. (Bottom gel) Restriction enzyme digestion with *Eco*RI of these PCR products generates two fragments of 1.6 kb and 0.6 kb (2.3 kb amplicon) or 2.4 kb and 0.6 kb (3 kb amplicon) demonstrating the specificity of PCR products. (C) PCR to screen cell lines excised for the BsR gene cassette by expressing the Cre-recombinase. PCR with primers #3 and #4 produces an expected 2 kb product only when the BsR gene cassette has been excised. Gel showing the PCR amplification products of 2xFIV+BsR containing Blasticidin as a negative control (Lane 1), 2xFIV (Lanes 2,3), 2xFIVmut (Lanes 4,5), 4xFIV-one side (Lanes 6,7), IL2RΔβ^A^ (Lanes 8,9), and DT40 wild type (Lane 10). (D) To screen cell lines targeted with 2xFIV+BsR vector on one allele and 2xFIV+Puro vector on the other one, a PCR is performed with primers #1 and #5 that flank the insertion point. Thus, genomic DNA extracted from cell lines containing both alleles targeted do not amplify the product. Gel showing the PCR products amplified from genomic DNA from wild type DT40 (Negative control: Lane 1), 2XFIV+BsR with only one allele targeted (Lane 2), and 2XFIV+BsR targeted on one allele and 2xFIV+Puro vector on the other one (Lanes 3,4). DT40 wild type cell line and one allele targeted 2xFIV+BsR amplified a 2.3 kb product, whereas both allele targeted cell lines did not amplify the expected product. (M, DNA marker; *, non-specific PCR product).
(TIF)

Table S1 Recapitulation of the constructs used and their effect on replication timing. The first column shows the different constructs used. The second column shows the average of late and early shifts for each construct. Finally, in the third column constructs are classified into four categories of replication timing shifts.
(EPS)

Table S2 Primer pairs used for quantitative PCR.
(TIF)

## Abbreviations

CGI: CpG island
ChIP: chromatin immunoprecipitation
NS: nascent strands
PI: propidium iodide
SNS: short nascent strands
TDP: timing decision point
USF: upstream stimulatory factor
WGA: whole-genome amplification
