# Supplementary figures and images for: USF Binding Sequences from the HS4 Insulator Element Impose Early Replication Timing on a Vertebrate Replicator
Source: PLoS Biol. 2012 Mar 6;10(3):e1001277. doi: 10.1371/journal.pbio.1001277 (PMC3295818; doi:10.1371/journal.pbio.1001277)

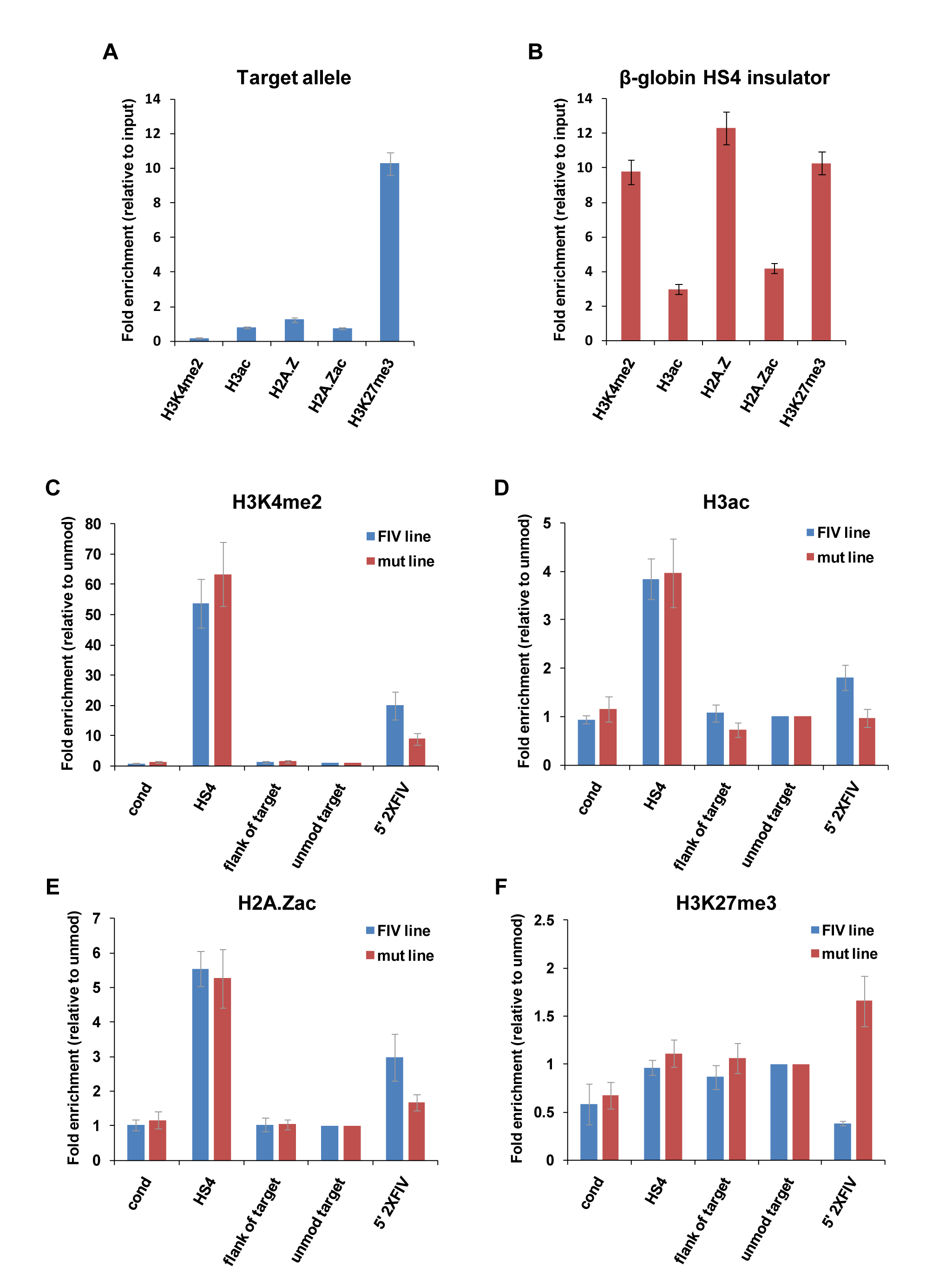

Supplement: Figure S1 — Transgenic USF binding sites recruit active histone modifications. Native chromatin immunoprecipitation analysis of histone modifications at DT40 loci following transgene integration. (A and B) Histone modification enrichments at the unmodified target allele (A) compared to the endogenous HS4 insulator element (B) as a reference (relative to input DNA). (C–F) Histone modifications in cells containing IL-2R transgenes that are flanked by wild type (blue bars) or mutant (red bars) FIV USF sites (as described in Figures 6B and 7A, respectively). The levels of H3K4me2, H3K9acK14ac, H2A.ZK4acK7acK11ac, and H3K27me3 relative to the unmodified target allele are shown in panels C to F, respectively. The levels of each modification at the transgenic FIV sites are shown, compared with those at the endogenous HS4 element and the condensed chromatin region upstream of the β-globin locus. (TIF) [file pbio.1001277.s001.tif]

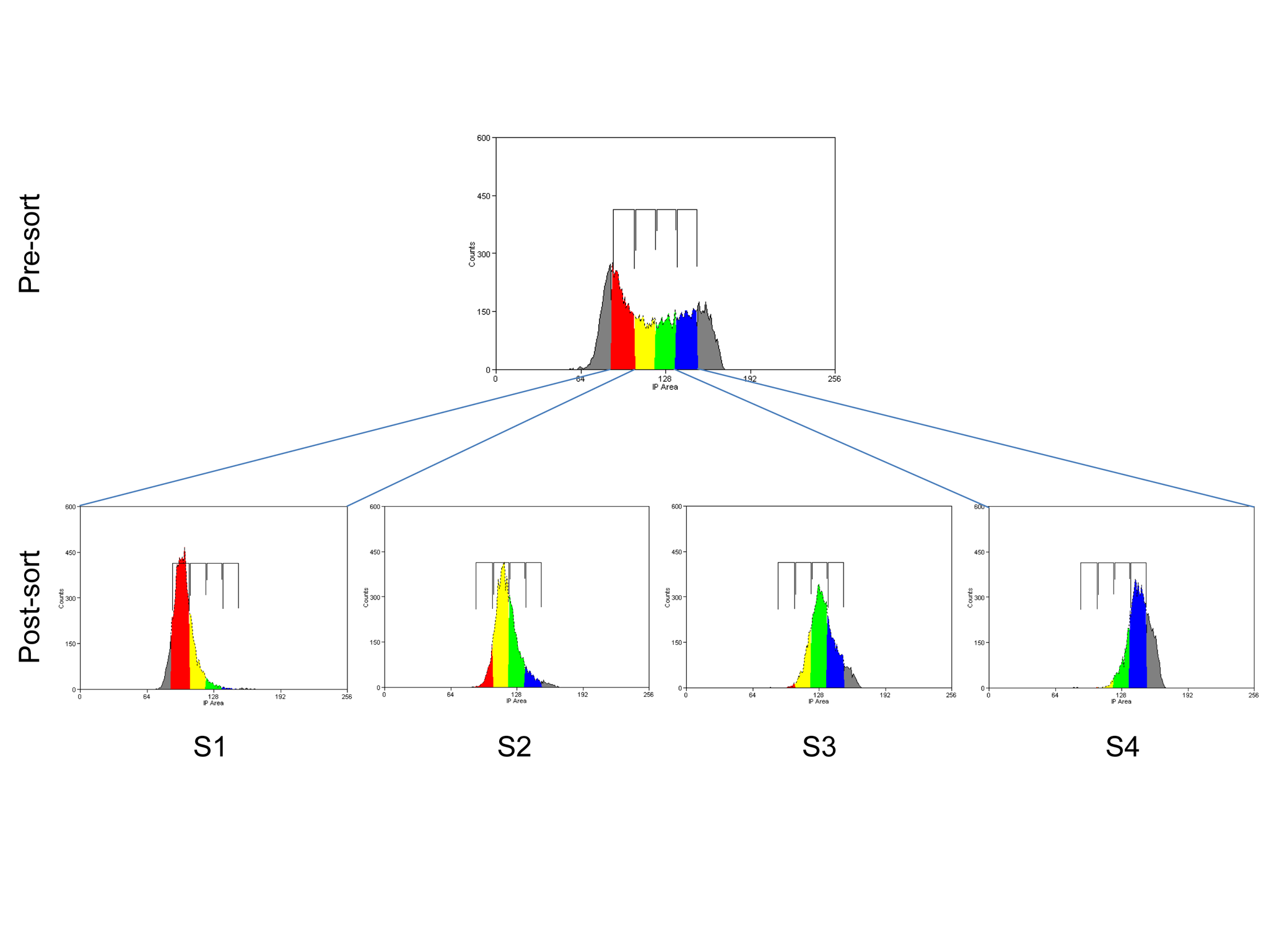

Supplement: Figure S3 — Analysis of the quality of the post-sorted fractions at the cellular level. The pre-sort (top) and post-sort cells (bottom, S1 to S4) were stained with PI and analyzed by flow cytometry. (TIF) [file pbio.1001277.s003.tif]

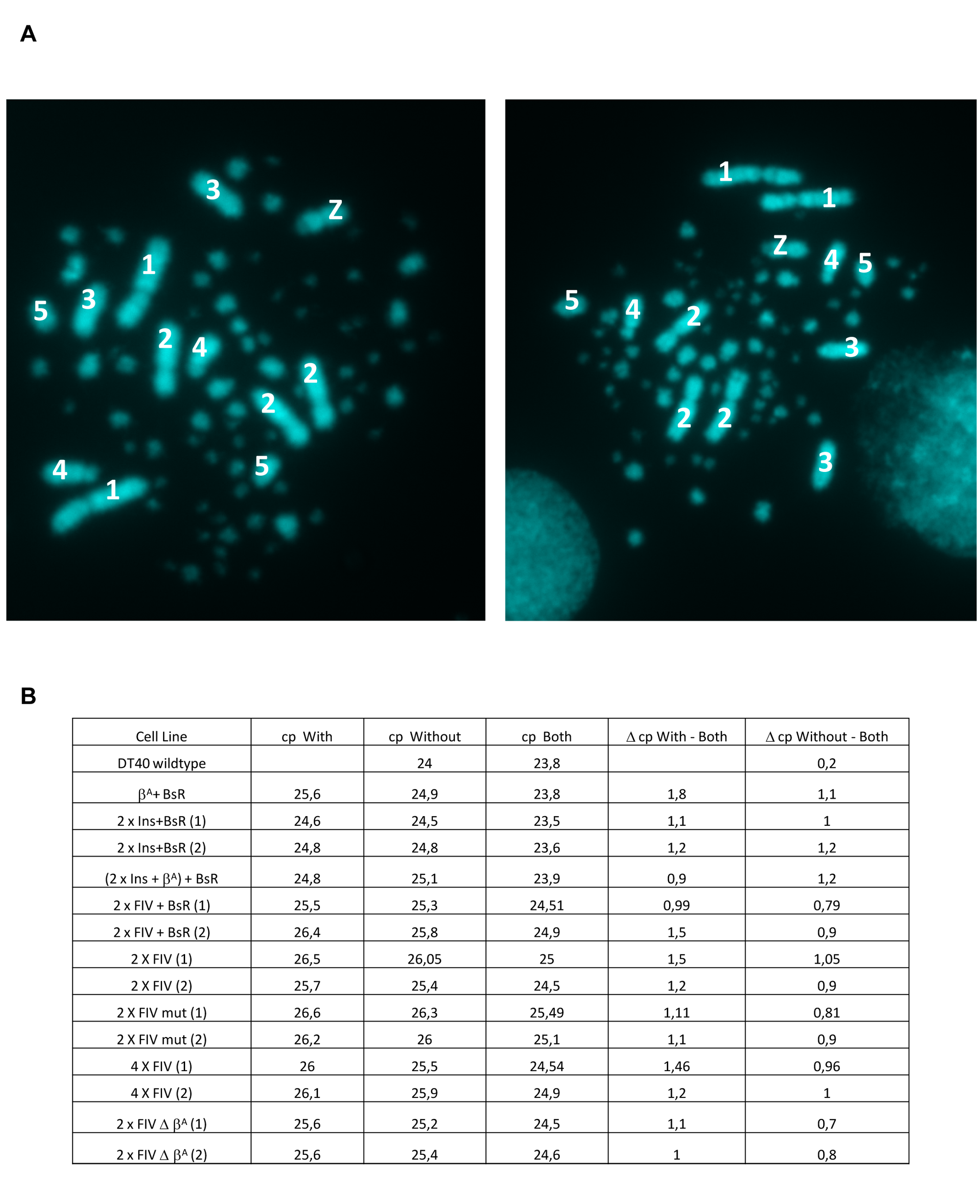

Supplement: Figure S4 — Karyotype analysis of the wild-type DT40 cell line used for every construct and validation by qPCR analysis of single targeted insertion of the transgene. (A) The figure shows two examples of a chromosomal spread. Fifteen chromosomal spreads were used to score the number of total chromosomes and especially the macrochromosomes, which are clearly distinguishable from each other. Consistent with the previous reports, we have scored 80 total number of chromosomes in DT40 cells used in this study [53]. A typical DT40 karyotype comprising the clearly distinct 11 autosomal macrochromosomes and one Z sex-chromosome (2Gga-1, 3Gga-2, 2Gga-3, 2Gga-4, 2Gga-5, 1Gga-Z) has been observed. (B) The table shows qPCR results obtained with genomic DNA extracted from clones selected for experiments shown in Figures 4– 8 (description of the clones is shown in Table S1). For each clone, 4 ng of genomic DNA was amplified with the three primer pairs (With, Without, and Both) described in Figure 3. Amplification was also performed in parallel on wild type DT40 genomic DNA with primer pairs Without and Both. The crossing point (cp) was obtained by using the second derivative maximum method described in the LightCycler Software (Roche). Each primer pair has a PCR efficiency around 95%; therefore, the difference in the crossing point (cp) between one and two copies should approximate one cycle. Analysis of transgene copy number shows that for most of the clones, a difference of one cycle is observed between the primer pair With (one copy) and Both (two copies). Three clones have a difference ≥1.5 due to slight variations in the efficiency of this primer pair that we have repeatedly observed. In any case this difference does not account for multiple copy insertion. When comparing the number of copies of the region containing the site of integration with a region located nearby, we also observed a difference of one cycle in every clone in contrast to the wild type DT40 cell line. This result con [file pbio.1001277.s004.tif]

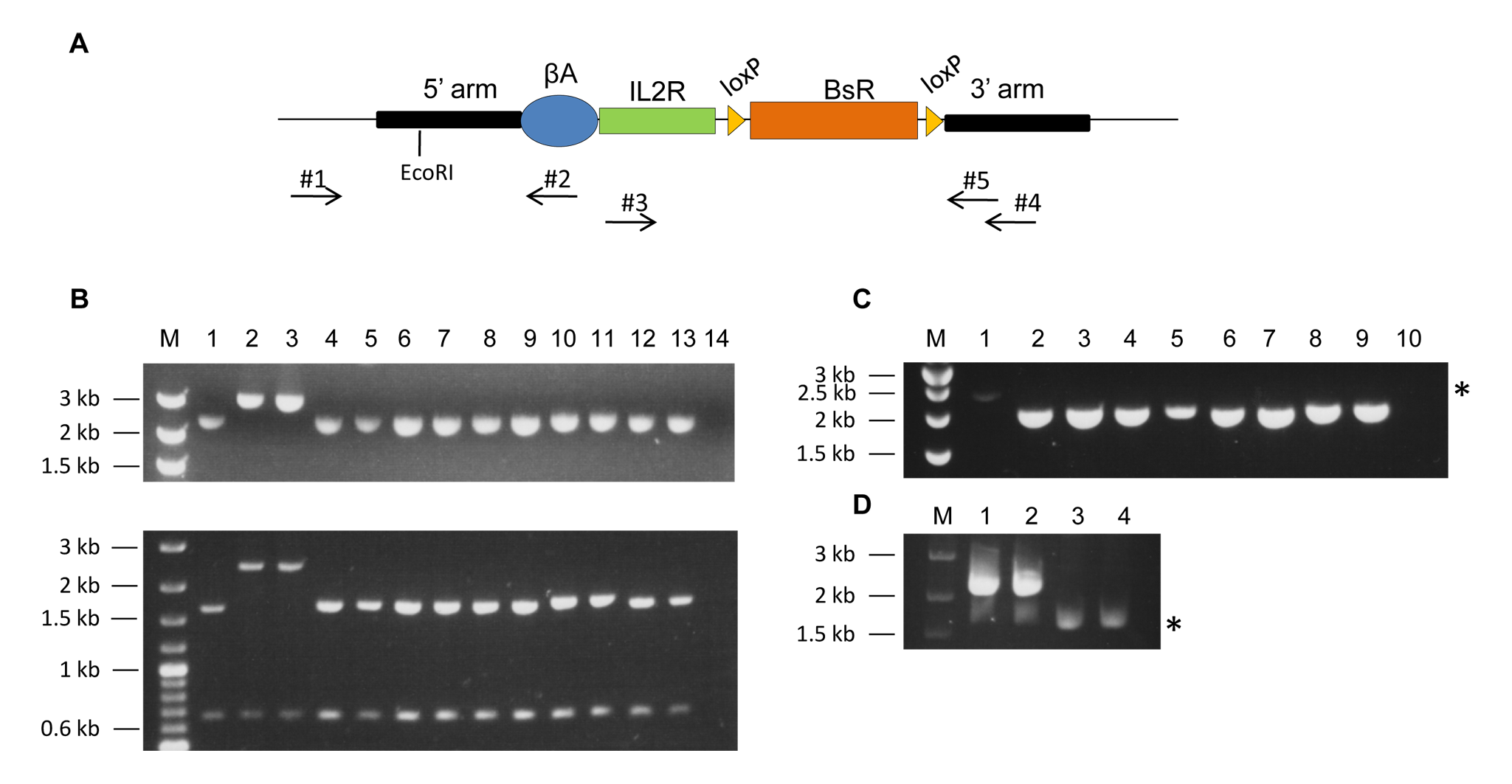

Supplement: Figure S5 — PCR validation of clones selected for homologous recombination. (A) Schematic diagram showing genomic region with a site-specific integrated construct. 5′ and 3′ arms of the targeted vector are shown as black boxes. A construct with βA origin (blue) linked to the IL-2R transgene (green) and Blasticidin (BsR) gene cassette (orange) flanked by loxP sites (yellow triangles) is shown. Arrows represent primers used for the analysis of integration of constructs by homologous recombination and correct excision of BsR. (B–D) Agarose gels showing PCR products. (B) PCR products amplified with primer #1 and #2 for testing the correctly integrated cell lines of βA+BsR (Lane 1), 2xIns+BsR (Lane 2), (2xIns+βA)+BsR (Lane 3), 2xFIV+BsR (Lanes 4,5), 2xFIV (Lanes 6,7), 2xFIVmut (Lanes 8,9), 4xFIV-one side (Lanes 10,11), double insertion lines with 2xFIV+BsR and 2xFIV+Puro (Lanes 12,13), and DT40 wild type as negative control (Lane 14). PCRs from genomic DNA extracted from correctly integrated cell lines should give a 2.3 kb product, except those containing 2xIns (Lanes 2 and 3), which should give a 3 kb product. (Bottom gel) Restriction enzyme digestion with EcoRI of these PCR products generates two fragments of 1.6 kb and 0.6 kb (2.3 kb amplicon) or 2.4 kb and 0.6 kb (3 kb amplicon) demonstrating the specificity of PCR products. (C) PCR to screen cell lines excised for the BsR gene cassette by expressing the Cre-recombinase. PCR with primers #3 and #4 produces an expected 2 kb product only when the BsR gene cassette has been excised. Gel showing the PCR amplification products of 2xFIV+BsR containing Blasticidin as a negative control (Lane 1), 2xFIV (Lanes 2,3), 2xFIVmut (Lanes 4,5), 4xFIV-one side (Lanes 6,7), IL2RΔβA (Lanes 8,9), and DT40 wild type (Lane 10). (D) To screen cell lines targeted with 2xFIV+BsR vector on one allele and 2xFIV+Puro vector on the other one, a PCR is performed with primers #1 and #5 that flank the insertion point. Thus, genomic DNA extracted from cell line [file pbio.1001277.s005.tif]

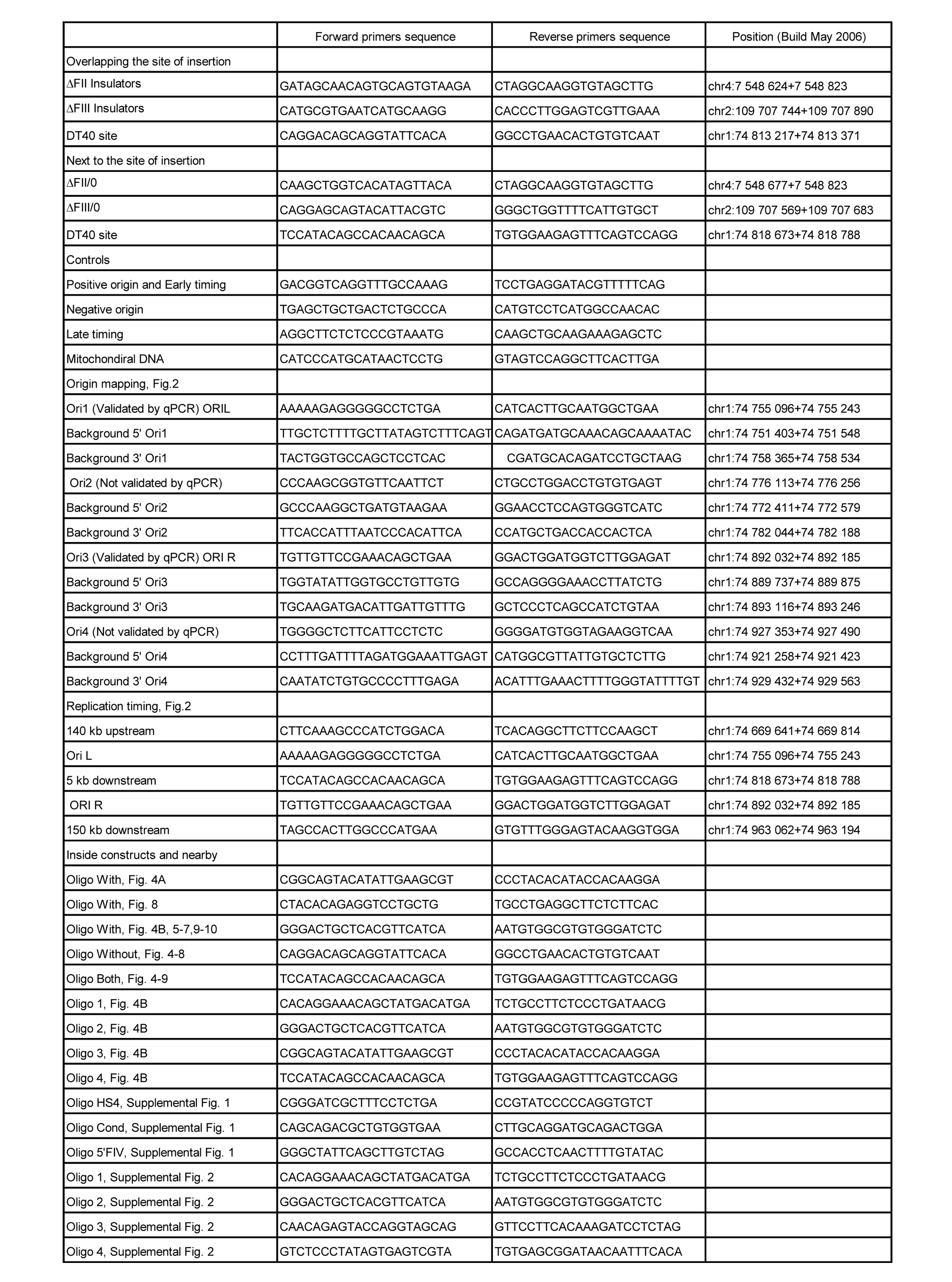

Supplement: Table S2 — Primer pairs used for quantitative PCR. (TIF) [file pbio.1001277.s007.tif]
